# Comparative genomics of the Western Hemisphere soft tick-borne relapsing fever borreliae highlights extensive plasmid diversity

**DOI:** 10.1186/s12864-022-08523-7

**Published:** 2022-05-31

**Authors:** Alexander R. Kneubehl, Aparna Krishnavajhala, Sebastián Muñoz Leal, Adam J. Replogle, Luke C. Kingry, Sergio E. Bermúdez, Marcelo B. Labruna, Job E. Lopez

**Affiliations:** 1 Department of Pediatrics, Baylor College of Medicine, Houston, TX USA; 2 Department of Molecular Virology and Microbiology, National School of Tropical Medicine, Baylor College of Medicine, Houston, TX USA; 3 Departamento de Ciencia Animal, Facultad de Ciencias Veterinarias, Universidad de Concepción, Concepción, Chile; 4 Division of Vector-Borne Diseases, Centers for Disease Control and Prevention, Fort Collins, CO USA; 5 Medical Entomology Department, Gorgas Memorial Institute for Health Research, Panamá City, Panamá; 6 Departamento de Medicina Veterinária Preventiva E Saúde Animal, Faculdade de Medicina Veterinária E Zootecnia, Universidade de São Paulo, São Paulo, Brazil

**Keywords:** Relapsing fever, *Borrelia*, Comparative genomics, Plasmids, Microbial genomics, Long-read sequencing

## Abstract

**Background:**

Tick-borne relapsing fever (TBRF) is a globally prevalent, yet under-studied vector-borne disease transmitted by soft and hard bodied ticks. While soft TBRF (sTBRF) spirochetes have been described for over a century, our understanding of the molecular mechanisms facilitating vector and host adaptation is poorly understood. This is due to the complexity of their small (~ 1.5 Mb) but fragmented genomes that typically consist of a linear chromosome and both linear and circular plasmids. A majority of sTBRF spirochete genomes’ plasmid sequences are either missing or are deposited as unassembled sequences. Consequently, our goal was to generate complete, plasmid-resolved genomes for a comparative analysis of sTBRF species of the Western Hemisphere.

**Results:**

Utilizing a *Borrelia* specific pipeline, genomes of sTBRF spirochetes from the Western Hemisphere were sequenced and assembled using a combination of short- and long-read sequencing technologies. Included in the analysis were the two recently isolated species from Central and South America, *Borrelia puertoricensis* n. sp. and *Borrelia venezuelensis*, respectively. Plasmid analyses identified diverse sequences that clustered plasmids into 30 families; however, only three families were conserved and syntenic across all species. We also compared two species, *B. venezuelensis* and *Borrelia turicatae*, which were isolated ~ 6,800 km apart and from different tick vector species but were previously reported to be genetically similar.

**Conclusions:**

To truly understand the biological differences observed between species of TBRF spirochetes, complete chromosome and plasmid sequences are needed. This comparative genomic analysis highlights high chromosomal synteny across the species yet diverse plasmid composition. This was particularly true for *B. turicatae* and *B. venezuelensis,* which had high average nucleotide identity yet extensive plasmid diversity. These findings are foundational for future endeavors to evaluate the role of plasmids in vector and host adaptation.

**Supplementary Information:**

The online version contains supplementary material available at 10.1186/s12864-022-08523-7.

## Introduction

Relapsing fever (RF) is a globally endemic yet neglected vector-borne disease caused by spirochetal bacteria of the *Borrelia* genus [1]. RF spirochetes are vectored by the human body louse, and both hard (ixodid) and soft (argasid) ticks. Tick-borne RF (TBRF) typically presents in humans with recurring fever and flu-like symptoms; however, neurological complications and death can occur [2]. The disease significantly impacts the impoverished in resource poor countries, while evidence suggests that TBRF spirochetes and their vectors are emerging in densely populated regions in the southwestern United States [3–8].

There are seven species of Western Hemisphere soft-tick-borne relapsing fever (WHsTBRF) spirochetes where laboratory isolates have been reported. These include *Borrelia anserina*, *Borrelia coriaceae*, *Borrelia hermsii*, *Borrelia parkeri*, *Borrelia turicatae*, *Borrelia puertoricensis* n. sp*.*, and *Borrelia venezuelensis* [9–16]. *Borrelia coriaceae, B. hermsii, B. turicatae,* and *B. parkeri* are distributed throughout North America [12, 17], while *B. anserina* has a global distribution [18]. Furthermore, laboratory isolates of *B. puertoricensis* and *B. venezuelensis* were only cultured within the last three years and originate from Panama and Brazil, respectively [9, 15]. Interestingly, a phylogenetic analysis of *B. venezuelensis* using the *flaB, rrs,* and *glpQ* loci indicated that this species formed a monophyletic group with *B. turicatae* with the two species sharing > 99% nucleotide identity [15]. The vast range difference between *B. turicatae* and *B. venezuelensis,* their different tick vectors, and the need for completed genomes spurred our interest to perform a genomic comparison between WHsTBRF spirochetes.

Borrelial genomes (Lyme and RF spirochetes) are small (~ 1.5 Mb) but exceptionally complex and distributed across many replicons [19, 20]. Their genomes typically consist of a linear chromosome and linear and circular plasmids. The linear replicons make up most of a genome. The number of plasmids in a given genome can range from five to 23 [21–23]. Due to the complex nature of borrelial genomes, particularly the covalently closed linear plasmids and repetitive elements, next-generation short-read DNA sequencing technologies (e.g. Roche 454, Illumina, Ion Torrent) have had difficulties in producing complete genomes [24]. The advent of third generation sequencing technologies (e.g. PacBio and Oxford Nanopore Technologies) opened new avenues for genome sequencing and assembly [25, 26]. However, compared to other bacteria, borrelial genomes require substantially more manual effort to resolve the plasmids. As a result, the plasmids of many publicly available borrelial genomes are highly fragmented across many contigs.

Since the first borrelial genome was completed in 1997 with *Borreliella* (*Borrelia*) *burgdorferi*, there have been 205 Lyme disease (LD) spirochete genomes deposited on GenBank compared to 44 TBRF genomes. Half of the TBRF genomes are of the hard tick-borne species, *Borrelia miyamotoi* [27]. Furthermore, the chromosomes of *B. anserina*, *B. coriaceae*, *B. hermsii*, *B. parkeri*, and *B. turicatae* have been assembled, and analyses between *B. hermsii* and *B. turicatae* indicate that they are largely collinear with similar gene content [28]. Thus, we reason that the chromosomes alone do not explain the diversity in vector competence and specificity, host reservoir adaptation, and pathogenesis seen in the WHsTBRF spirochete clade; plasmids likely play a significant role as well [1, 18, 29–32].

In this study, we generated complete, plasmid-resolved genomes of available isolates from the WHsTBRF spirochete clade and performed a comparative genomics analysis between these species. We focused on *B. anserina* BA2, *B. coriaceae* Co53, *B. hermsii* DAH and YOR, *B. parkeri* SLO, *B. turicatae* 91E135 and BTE5EL, *B. puertoricensis* n. sp. SUM [9], and *B. venezuelensis* RMA01 because they are either commonly used laboratory isolates, or their genomes have not been reported. We hypothesized that WHsTBRF spirochete genomes consist of a syntenic chromosome with plasmids grouping into distinct families, which are diverse between species. To test this hypothesis, analyses were performed to determine the relatedness of these strains by using whole genome methods including average nucleotide identity (ANI) analysis and phylogenomic analyses. Pangenome analysis was also performed to investigate gene content differences across all species. Plasmid diversity and similarities were evaluated as well through phylogenetic and dot plot analyses. We concluded our efforts by comparing the genomes of two species, *B. venezuelensis* and *B. turicatae*, which have been isolated from two different *Ornithodoros* species but were reported to be closely related genetically [15]. This work is important in progressing efforts to identify the molecular mechanisms of pathogenesis, vector biology, and the evolution and ecology of RF spirochetes and the *Borreliaceae*.

## Results

### Genomic evaluation of WHsTBRF spirochetes

Within the Western Hemisphere, seven TBRF species have been identified and isolated [15, 29], and a representative genome from each species was evaluated. The analysis also included a representative from each of the *B. hermsii* genomic groups (genomic group I: DAH, genomic group II: YOR) [33]. Furthermore, we evaluated a human isolate of *B. turicatae* (BTE5EL) and a commonly used laboratory isolate (91E135) (Table 1).

**Table 1:** *Borrelia* isolates used in this study

Species	Isolate	Geographic Location	Origin	Tick Vector	Reference
*B. anserina*	BA2	USA	Chicken blood	*Argas* spp.	[10]
*B. hermsii*	YOR	California, USA	Human blood	*Ornithodoros hermsi*	[11]
*B. hermsii*	DAH	Washington, USA	Human blood	*O. hermsi*	[16]
*B. coriaceae*	Co53	California, USA	*Ornithodoros coriaceus*	*O. coriaceus*	[12]
*B. puertoricensis* n. sp.	SUM	Panama	*Ornithodoros puertoricensis*	*O. puertoricensis*	[9]
*B. parkeri*	SLO	California, USA	*Ornithodoros parkeri*	*O. parkeri*	unpublished
*B. venezuelensis*	RMA01	Maranhão, Brazil	*Ornithodoros rudis*	*O. rudis*	[15]
*B. turicatae*	91E135	Texas, USA	*Ornithodoros turicata*	*O. turicata*	[13]
*B. turicatae*	BTE5EL	Texas, USA	Human blood	*O. turicata*	[14]

**Fig. 1:**
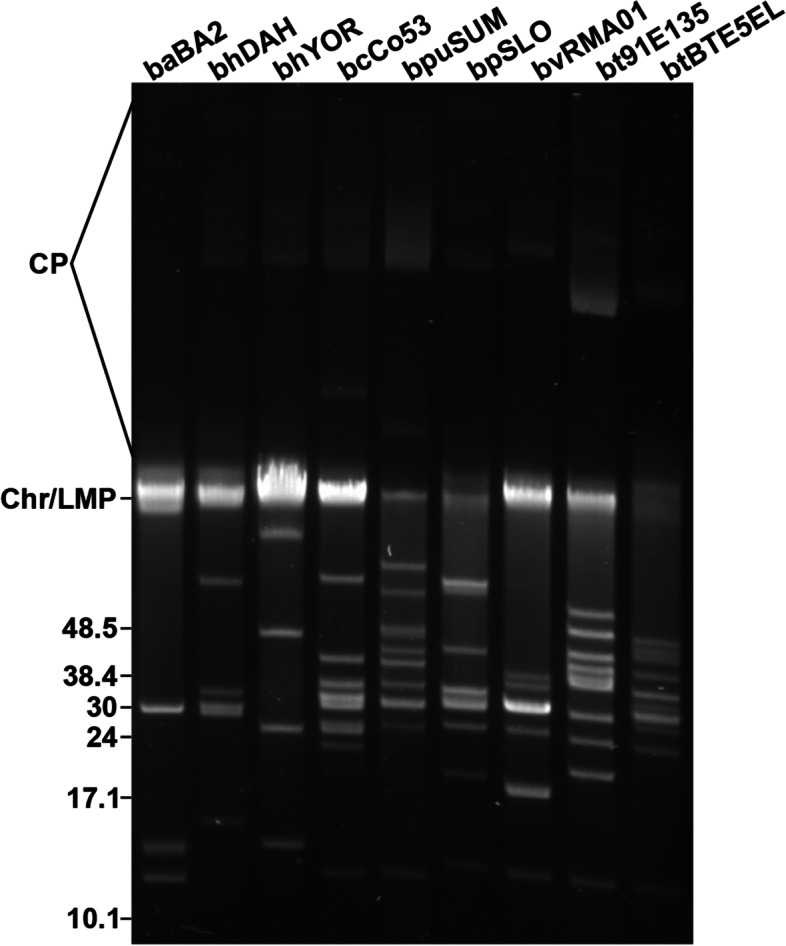
Pulsed-field gel electrophoresis (PFGE) of WHsTBRF species. PFGE was performed on genomic DNA of each isolate to demonstrate the different plasmid profiles both between and within species. Isolate names have been abbreviated as follows: baBA2, *B. anserina* BA2; bhDAH, *B. hermsii* DAH; bhYOR, *B. hermsii* YOR; bcCo53, *B. coriaceae* Co53; bpuSUM, *B. puertoricensis* n. sp. SUM; bpSLO, *B. parkeri* SLO; bvRMA01, *B. venezuelensis* RMA01; bt91E135, *B. turicatae* 91E135; btBTE5EL, *B. turicatae* BTE5EL. The numbers to the left of the gel image correspond to DNA size in kilobases. Chr/LMP corresponds to the co-migration of the chromosome and linear megaplasmid, respectively. CP refers to circular plasmids. The full-length, uncropped gel is presented in Additional File 1: Fig S1

To aid in genome assembly of plasmids, we performed pulsed-field gel electrophoresis of genomic DNA isolated from each strain, which showed varying complexity and exemplified the variation in plasmid sizes (Fig. 1). Genomic profiles indicated that *B. anserina* BA2 was a relatively simple genome displaying approximately four plasmids while *B. coriaceae* Co53 and *B. turicatae* 91E135 were more complex with ~ 10 and ~ 11 visible linear plasmids, respectively. Furthermore, the high molecular weight bands on pulsed-field gels represent the presence of circular plasmids [33]. Interestingly, circular plasmids have been reported to be absent in *Borrelia parkeri* [34], but this analysis suggested evidence of circular plasmid(s) in the SLO isolate (Fig. 1).

Genome assembly and annotation results are summarized in Additional File 2: Table S1. The dataset contained a linear chromosome for each species and a total of 128 plasmids comprised of 94 linear and 34 circular plasmids. The average number of plasmids per genome was 13.9 (range: 5–23). The average circular to linear plasmid ratio was 0.35 (range: 0.0–0.83). The smallest genome was *B. anserina* BA2 (1.05 Mb), which contained the least number of plasmids (four) and no circular plasmids. The largest genome was that of *B. puertoricensis* n. sp. SUM (1.87 Mb), which also contained the most circular plasmids, 10. The strain with the largest number of plasmids, overall, was *B. coriaceae* Co53 (18 linear plasmids and five circular). Interestingly, sequencing and assembly of *B. parkeri* SLO confirmed the presence of circular plasmids. We identified multiple circular plasmids in all genomes analyzed except *B. anserina* BA2 and *B. venezuelensis* RMA01. *Borrelia anserina* BA2 contained no circular plasmids while *B. venezuelensis* RMA01 contained only one circular plasmid (cp28). Collectively, a variety of differences in genome composition existed across the genomes of the WHsTBRF clade as exemplified by the number of plasmids, and differences in both length and topology.

### ANI and phylogenomic analyses

ANI is a robust method for determining whole genome sequence identity by analyzing orthologous regions between genomes [35, 36]. Clear species boundaries have been shown for prokaryotes at intraspecies values > 95% and interspecies values < 83% [37]. Using a North American hard tick-borne relapsing fever spirochete (*B. miyamotoi* CT13-2396) as an outgroup, we calculated the ANI in an all-vs-all comparison of our genome assemblies (Additional File 1: Table S2). We observed an ANI between both *B. turicatae* isolates (91E135 and BTE5EL) of ~ 99%, which is substantially higher when compared to the ANI of the isolates representing both genomic groups of *B. hermsii* (DAH and YOR) at ~ 95.7%. Surprisingly, *B. venezuelensis* RMA01 and *B. turicatae* 91E135 and BTE5EL genomes indicated high ANI (~ 98.5%) despite being isolated from different *Ornithodoros* species located ~ 6,800 km apart from each other.

**Fig. 2:**
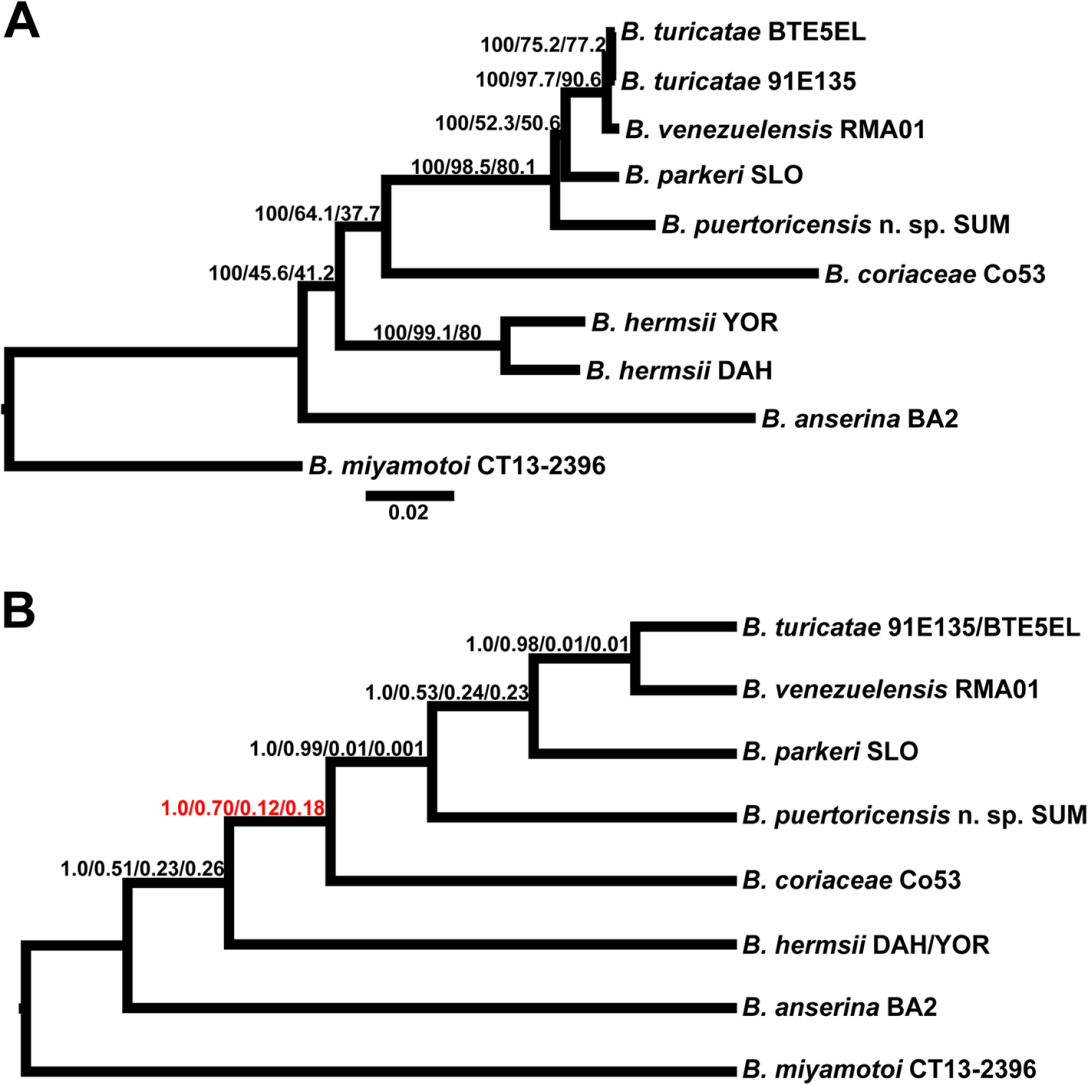
Phylogenomic analyses A maximum likelihood species tree was inferred from a concatenation of 650 single-copy core genes (720,532 nucleotide sites). **A** The tree was generated using an edge-linked proportional partition model with 1,000 ultra-fast bootstraps, and a subsequent concordance factor analysis was performed. Branch support is reported as ultra-fast bootstrap/gCF/sCF (see text for description of gCF and sCF). The scale bar is representative of substitutions per site. A coalescence-based cladogram was inferred from the 650 genes used in **A**. **B** The cladogram branch supports are reported as local posterior probability, major quartet frequency, alternative quartet frequency 1, and alternative quartet frequency 2. Red branch supports indicate alternative quartet frequencies that are significantly unequal (*p* < 0.001) indicating that incomplete lineage sorting alone does not explain that topology

We investigated the phylogenetic relationships of the WHsTBRF spirochetes using two different species tree inference methods, concatenation and coalescence. By using the Panaroo pipeline, we identified 741 single-copy core genes between the WHsTBRF clade and *B. miyamotoi* CT13-2396 and performed phylogenomic analysis with this data set [38]. We tested these genes for substitution saturation as this can negatively impact the accuracy of phylogenomic analysis [39]. Saturation analysis determined that 91 genes were likely saturated and thus were removed from this dataset (Additional File 3). A maximum likelihood species tree was inferred from a concatenation of 650 single-copy core genes (720,532 total nucleotide sites) using an edge-linked proportional partition model with nucleotide sequences (Fig. 2A). The species tree of single-copy core genes recapitulated previously published tree topologies with high bootstrap support (100% in all cases) [40, 41]. To further determine if our tree was valid, we used concordance factor (CF) analysis. This analysis is important when using large datasets for phylogenomic species tree inference as there can be high bootstrap support for wrong topologies [42]. While the concatenation-inferred species tree showed high bootstrap value support for all branches, there was varying gene CF (gCF) and site CF (sCF) support. The *B. hermsii* and *B. parkeri* branches had low gCF and sCF support. We further observed that the phylogenomic analysis supported, through both bootstrap and gCF and sCF, that *B. venezuelensis* is a highly related sister taxon to *B. turicatae*. To account for potential gene tree discordance due to divergent coalescent histories we also inferred a species tree using the ASTRAL tool [43].

The tree inferred by ASTRAL maintained the same topology as the concatenation tree and was well supported by local posterior probabilities at all branches (Fig. 2B). The quartet support of the ASTRAL tree also agreed with the gCF and sCF support seen in the concatenation tree. Interestingly, alternative quartet frequencies for the *B. coriaceae* branch were statistically different indicating that incomplete lineage sorting alone could not be attributed to driving this topology [44].

### Pangenome analysis

**Fig. 3:**
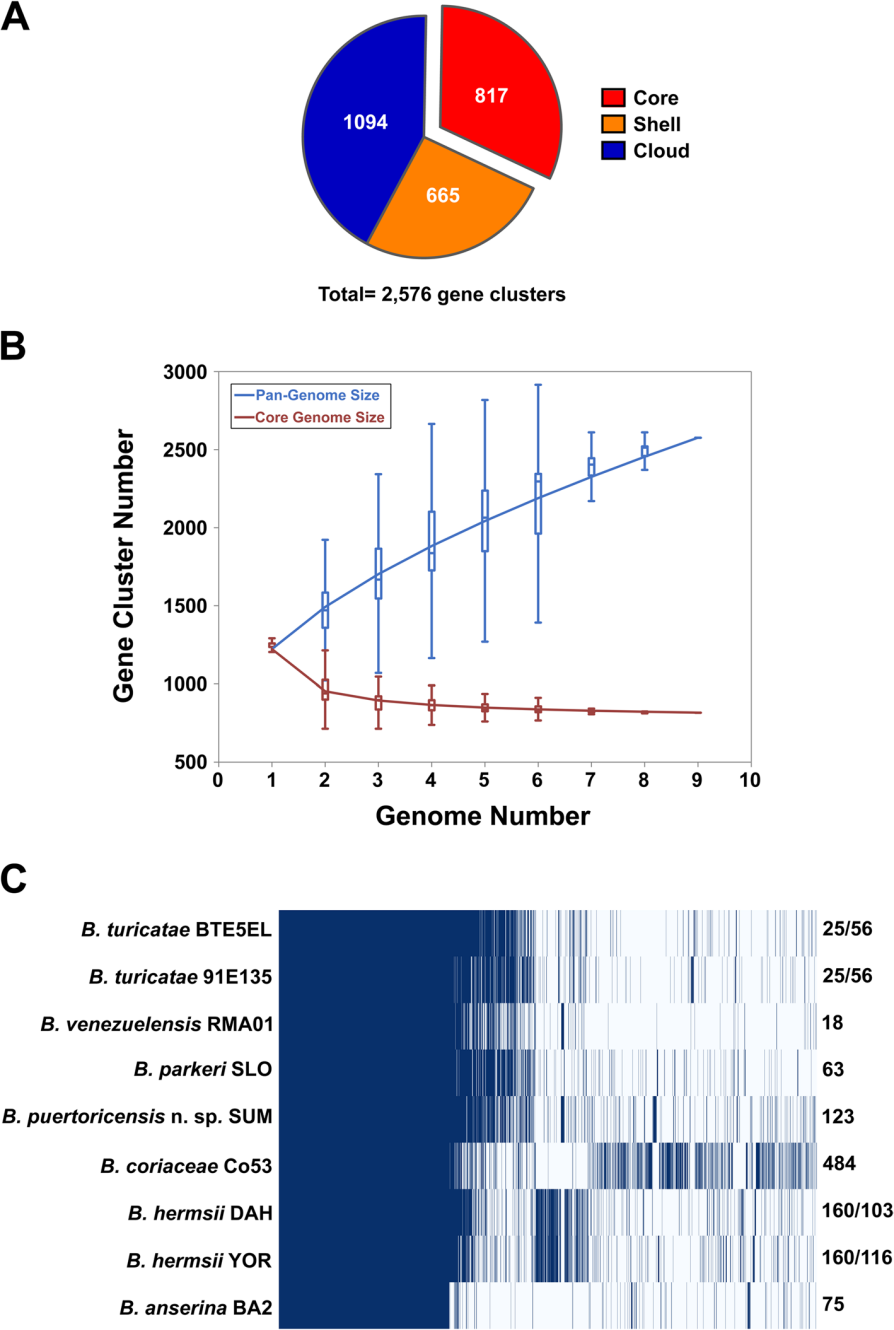
Pangenome analysis. The core genome in this analysis is defined as gene clusters present in all nine genomes. The shell genome is defined as gene clusters present in two to eight genomes. The cloud genome is defined as gene clusters present in only 1 genome (**A**). A graph of the pan- and core-genomes was generated with PanGP, which indicated that the WHsTBRF clade pangenome is open and stabilizes after four genomes (**B**). Also shown is the pangenome matrix (**C**)**.** The numbers on the right indicate the number of unique gene clusters for each isolate’s genome. In the case of multiple isolates per species (*B. hermsii* and *B. turicatae*) the species-specific unique gene clusters are reported first followed by the isolate-specific unique gene clusters (**C**). Taxa prefixes: btBTE5EL, *B. turicatae* BTE5EL; bt91E135, *B. turicatae* 91E135; bvRMA01, *B. venezuelensis* RMA01; bpSLO, *B. parkeri* SLO; bpuSUM, *B. puertoricensis* n. sp*.* SUM; bcCo53, *B. coriaceae* Co53; bhDAH, *B. hermsii* DAH; bhYOR, *B. hermsii* YOR; baBA2, *B. anserina* BA2

Pangenome analysis of the WHsTBRF clade estimated there to be a core genome of 817 gene clusters and an accessory genome of 1,759 gene clusters for a total pangenome size of 2,576 gene clusters (Fig. 3A). The core genome stabilized after the addition of approximately five genomes but the pangenome size continued to increase with each genome (Fig. 3B). The continued increase in pangenome size is most likely due the expansive paralogous gene families and plasmid diversity seen in borrelial species [22, 27]. The differences between species and the unique gene clusters they contained can be appreciated in the pangenome matrix plot (Fig. 3C). *Borrelia coriaceae* Co53 contributed the most unique gene clusters, 484, while *B. venezuelensis* RMA01 contributed the least, 18. The pangenome analysis indicated a diverse and open pangenome for the WHsTBRF clade and identified a sizeable amount of unique gene content in the *B. coriaceae* Co53 genome.

### Chromosome and plasmid sequence analyses

#### Chromosomal sequence analysis

Previously the *B. hermsii* and *B. turicatae* chromosomes were reported to be syntenic and collinear [28], and we evaluated this across all species of the WHsTBRF borreliae. We investigated the chromosomes of our dataset first by performing ANI on chromosomal sequences only (using *B. miyamotoi* CT13-2396 as an outgroup). We observed a wide degree of chromosome sequence identity in the WHsTBRF spirochetes from 85.92% to 99.44% between species (Additional File 2: Table S2**).**

We also assessed synteny in WHsTBRF clade’s chromosomes by aligning these sequences using the Mauve aligner [45]. This analysis indicated a single, large collinear block that showed no rearrangements among the different chromosomes (Additional File 1: Fig S2). With the lack of internal structural rearrangements and the wide variation in ANI, we investigated gene content differences between chromosomes using Panaroo analysis. Panaroo was used to determine gene presence or absence differences, and to determine the core genome gene clusters that resided on the chromosome. The majority of the core genome was found in the chromosome (94%, 767 gene clusters), and largely accounts for many of the housekeeping and essential gene functions (e.g. DNA replication and repair, translation, protein synthesis, etc.). Most of the chromosomal gene content was similar, with gene presence or absence differences shown in Additional File 2: Table S3. Of the 874 gene clusters identified during the chromosomal sequence analysis, 767 gene clusters (79% of chromosomal gene clusters) were present in all isolates. The remaining 107 gene clusters were differentially present between isolates. While analyzing the gene content of the chromosome sequences, we noticed structural differences on the telomeric ends.

Both the 5’ and 3’ chromosome telomeres lacked synteny and contained gene content that differed between species. Investigation of these sequences showed the chromosome telomeres to be identical to plasmid telomere sequence (Additional File 1: Fig S3), with the exception of *B. hermsii* whose 5’ chromosome telomere was not found in its plasmids. Of note, we identified the presence of a *variable major protein* (vmp) allele on the 5’ telomere of the *B. coriaceae* Co53 chromosome (*bcCo53_000002*). The *vmp* alleles are important for RF spirochete pathogenesis and exist in two different but functionally synonymous types, variable large and variable small proteins [2, 46–49]. The *bcCo53_000002* allele was intact and annotated as a variable large protein. Vmp alleles have not been previously reported on RF spirochete chromosomes. While sequences of RF spirochete chromosomes have been available for some time, many are missing the telomeric ends of the chromosome. Our work indicates that while most of the chromosome is syntenic, gene exchange and accumulation between chromosome and plasmid has occurred in the telomeres.

#### Plasmid sequence analysis

**Fig. 4:**
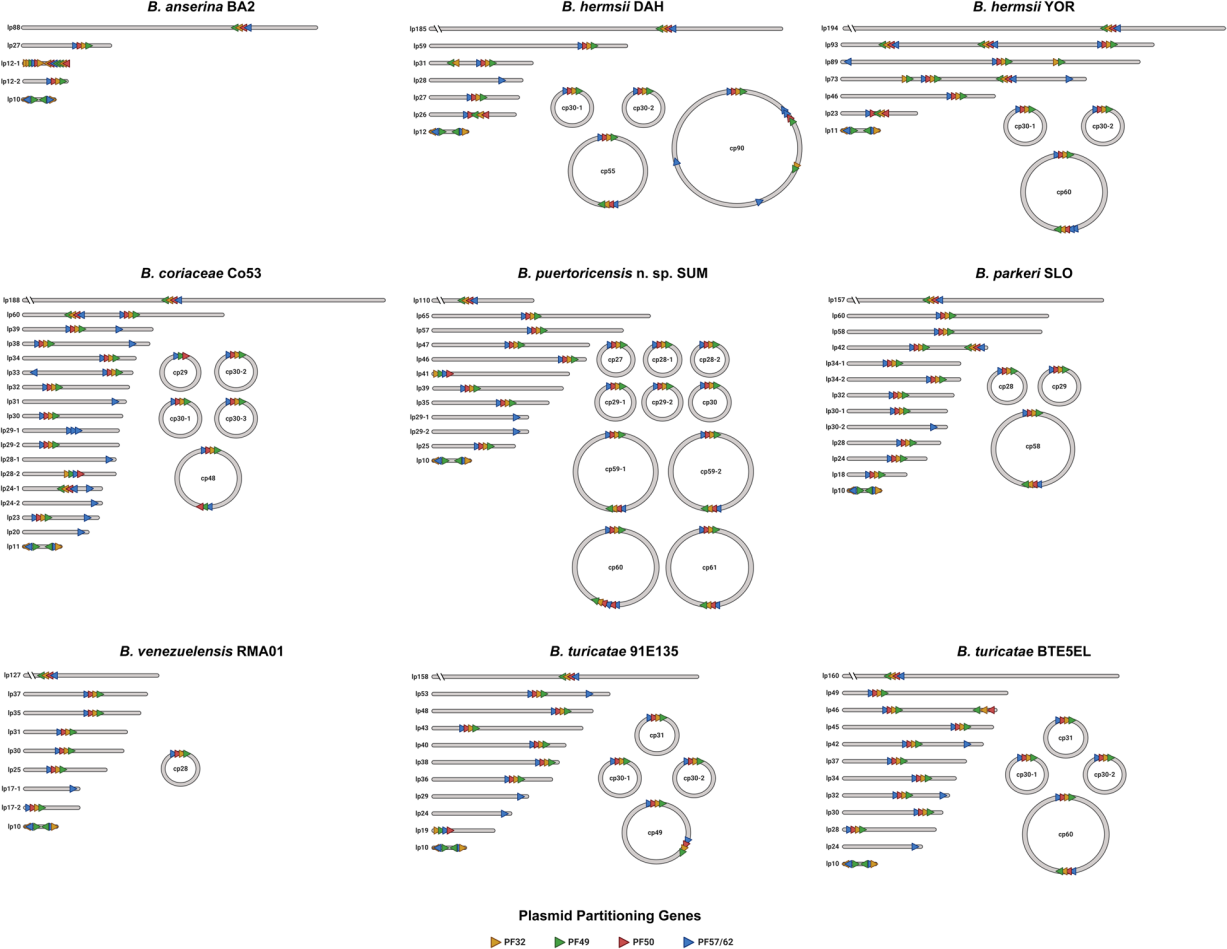
Plasmid repertoires and approximate locations of plasmid partitioning genes. The proportionate sizes of the plasmids found in each isolate are graphically depicted. The replicons are named based on topology (lp = linear plasmid, cp = circular plasmid) followed by the size to the nearest kilobase. The breaks in the megaplasmids, denoted by the discontinuity found on the left side of the megaplasmid, are of 80 kb except for *B. anserina* BA2 which was not broken. The approximate location of the PF32, PF49, PF50, and PF57/62 plasmid partitioning genes and their orientations are also shown with triangles facing right for positive-sense and left for negative-sense. This figure highlights the possibility of past fusion events on plasmids that have multiple plasmid partitioning gene(s) of the same type. Created with BioRender.com

With the generated genomes, we were positioned to analyze and compare the plasmid sequences of the WHsTBRF spirochetes. We determined the number and location of putative plasmid partitioning genes for all plasmids (Fig. 4). Plasmid partitioning genes are important for the stable, heritable maintenance of plasmids in bacteria. At least five *Borrelia* paralogous gene families have been identified as putative plasmid partitioning genes: PF32, PF49, PF50, PF57, and PF62 [50–52]. The PF57 and PF62 gene families have limited similarity to other genes outside of *Borrelia* but are thought to be functionally redundant and have been grouped together as PF57/62 [20, 50]. Analysis of plasmid partitioning genes showed 39 plasmids contained multiple sets or copies of these genes (Fig. 4). The presence of multiple copies of partitioning genes on the same replicon suggests plasmid fusion events as each plasmid should contain a unique set or copy of the plasmid partitioning genes [50]. However, because these genes are likely involved in plasmid compatibility, containing the same set of partitioning genes on multiple plasmids would be detrimental. Such an event would result in plasmid incompatibility resulting in plasmid loss in daughter cells [21, 51]. Consequently, we would expect plasmid partitioning genes to be divergent in nucleotide identity within the same genome. To further investigate the relationships between plasmids and the partitioning genes, we performed phylogenetic analysis.

Borrelial plasmids have been previously investigated and classified by phylogenetic analysis of PF32 loci [21, 22, 25], and we performed a similar analysis with our dataset (Additional File 1: Fig S4). However, we identified fourteen plasmids lacking a PF32 locus, so we performed a phylogenetic analysis of PF57/62 loci (Additional File 1: Fig S5), which was found in every plasmid in at least one copy. In these phylogenetic analyses, we included LD spirochete and *B. miyamotoi* CT13-2396 PF32 and PF57/62 loci (deposited genomes used are indicated in Additional File 2: Table S4) [22]. The PF32 loci phylogenetic analysis determined WHsTBRF spirochetes had a total of 30 plasmid clusters based on topology. Eleven of these plasmid clusters were related to plasmid-types found in LD spirochetes (Additional File 1: Fig S4). Seven plasmid clusters contained plasmids found in *B. miyamotoi* CT13-2396, while nine clusters did not contain a plasmid from either the LD spirochetes or *B. miyamotoi* CT13-2396 (Additional File 1: Fig S4). Within all plasmid clusters there were a variety of plasmid topologies and sizes. Due to this finding, adopting the nomenclature used in LD spirochete plasmid typing was not practical. Consequently, we designated each cluster as a plasmid family (“F” for family, F1-30) for reference in this and future analyses until more complete work can develop a harmonized nomenclature for New- and Old-World RF spirochete plasmids.

To validate the phylogenetic plasmid clusters, we performed sequence identity clustering of the PF32 loci. This analysis generally agreed with the phylogenetic analyses and demonstrated intra-cluster nucleotide sequence identities ≥ 85% (Additional File 4). There were five plasmid families where nucleotide sequence clustering of PF32 loci did not agree with the clustering in the phylogenetic analysis (F4, F5, F20, F21 and F28 families). Orphan PF32 loci that did not cluster with any plasmid family were also identified. Four orphan PF32 loci were found by phylogenetic clustering based on topology (*baBA2*_*000862*_lp12-1_ps, *bcCo53_001336*_lp24-1, *AXH25_RS04465*_cp2, and *btBTE5EL_001353*_lp32) (Additional File 1: Fig S4); however, only three of these loci were orphaned by the sequence identity clustering (*baBA2_000862*_lp12-1_ps, *AXH25_RS04465*_cp2, and *btBTE5EL_001353*_lp32) (Additional File 4).

Phylogenetic analysis of PF32 loci showed evidence of extensive inter–plasmid family fusions in both circular and linear plasmids. For example, multiple PF32 loci were found on the same plasmid but clustered in different plasmid families (e.g. bhYOR_lp93, bpSLO_lp42, bpuSUM_cp59-1, bt91E135_cp49) (Additional File 1: Fig S6). Plasmid fusion events have been described previously in borrelial genomes [20–22]; however, since PF32 genes are reportedly important for borrelial plasmid compatibility, no genome should contain two intact PF32 loci of the same family on different plasmids.

We identified a total of four occurrences in four assemblies where multiple copies of PF32 loci from the same family designation were found on different plasmids. Monomer and heterodimers of circular plasmids were the most common occurrences of the same PF32 loci on different plasmids in the same genome (*bhYOR_000915*_cp30-2 and *bhYOR_001000*_cp60, *bpSLO_000869*_cp28, *bpSLO_000910*_cp29, *bpSLO_000951*_cp58 and *bpSLO_000994*_cp58). We detected reads consistent with both length and sequence of monomeric and dimer forms, thus we included these replicons in the final assemblies. However, the long-term stability of such occurrences is unknown and we cannot rule out that these forms exist independently across the polyclonal population of cells.

There were two occurrences of similar PF32 loci on different linear plasmids in the same genome. The *B. hermsii* YOR lp73 and lp89 plasmids both had an F4 PF32 locus (*bhYOR_001322* and *bhYOR_001456,* respectively). Further investigation of these loci found that they were less than 50% the length of other PF32 loci in the F4 family (these were not predicted to be pseudogenes). These loci were immediately upstream of PF49 pseudogenes with no PF50 or PF57/62 loci near them. It is likely that *bhYOR_001322* and *bhYOR_001456* are remnants from a fusion event and are not functional, thus we posit this would not cause plasmid instability. The *B. venezuelensis* RMA01 lp35 and lp89 plasmids both had a similar F28 PF32 locus (*bvRMA01_001193* and *bvRMA01_001225*, respectively). These plasmids were nearly identical except for sequence near and including the 3’ telomere. This finding is part of a subsequent analysis of our dataset (manuscript in preparation). Interestingly, 13 linear plasmids only had the PF57/62 locus and no other plasmid partitioning genes (Fig. 4).

To account for the plasmids lacking a PF32 locus we performed phylogenetic analysis of the PF57/62 locus (Additional File 1: Fig S5). This analysis showed weak agreement with the PF32 phylogeny; however, phylogenetic clustering agreed for F6, F20, F27, and F28 plasmids. Interestingly, the 13 PF57/62-only plasmids clustered into a single monophyletic clade with three distinct subclades, where *B. coriaceae* Co53 formed a clade separate from the rest of the WHsTBRF spirochetes and *B. miyamotoi* CT13-2396. Linear PF57/62-only plasmids have been described before in *B. miyamotoi* and in LD spirochetes [20, 25]. The *B. miyamotoi* CT13-2396 PF57/62-only plasmids did not cluster with the WHsTBRF spirochete PF57/62-only plasmids. Lyme disease spirochete plasmids containing only PF57/62 genes (i.e. lp5) did not cluster with the WHsTBRF clade’s PF57/62-only plasmids suggesting they may be specific to TBRF spirochetes.

Dot plot analysis of the PF57/62-only plasmids demonstrated intra-clade relatedness and synteny for the non-*B. coriaceae* Co53 clades (Additional File 1: Fig S7). The non-*B. coriaceae* Co53 clades had presence of phage-related genes including a structural gene, *tape measure protein*, a part of the phage tail. The *B. venezuelensis* RMA01 lp17-1 plasmid had limited synteny to the other plasmids in the non-*B.coriaceae* Co53 clades and did not carry any discernable phage-related genes. The plasmids in the *B. coriaceae* Co53 clade were not syntenic and all but two (lp28-1 and lp29-1) carried *vmp* alleles. *Vmp* alleles were not seen in the other species’ PF57/62 clade. From these two clades several fusion events have occurred with other plasmids. Fusions appeared to have occurred in the *B. coriaceae* Co53 PF57/62-only plasmids but were not present in *B. puertoricensis* n. sp. SUM, *B. parkeri* SLO, or *B. venezuelensis* RMA01 plasmids (Additional File 1: Fig S8).

**Fig. 5:**
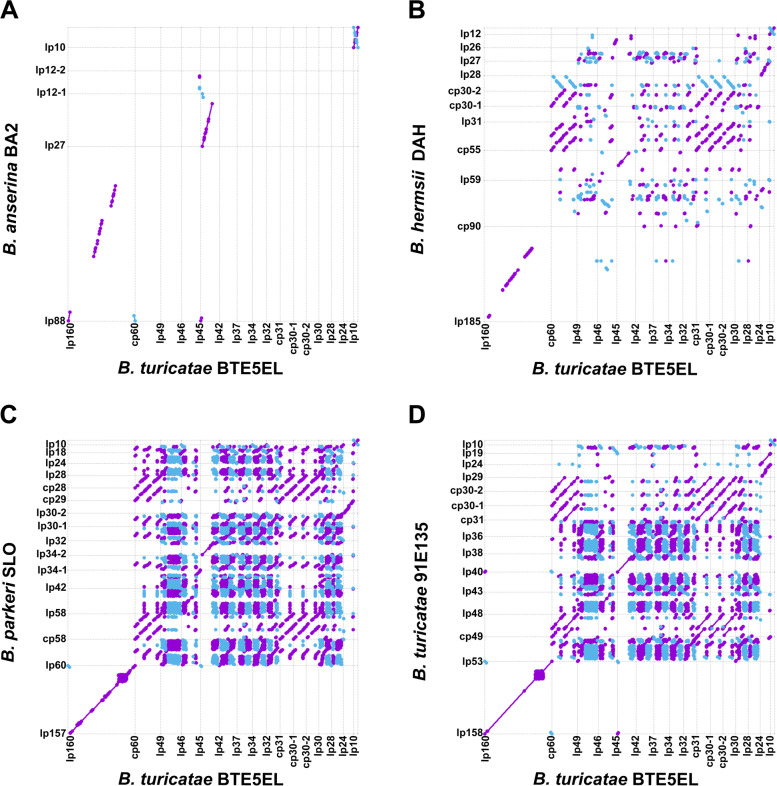
Representative plasmid dot plots of the WHsTBRF spirochete plasmid sequences. Dot plots were generated in a pairwise fashion of the WHsTBRF spirochete plasmids between isolates. Representative dot plots are shown between the most divergent species (*B. anserina* BA2 and *B. turicatae* BTE5EL, **A**), between *B. hermsii* DAH and *B. turicatae* BTE5EL (**B**) and *B. parkeri* SLO and *B. turicatae* BTE5EL (**C**), and isolates of the same species (*B. turicatae* 91E135 and BTE5EL, **D**). Synteny is represented by a purple line from the bottom left corner to the top right corner of each comparison. Inversions are indicated in blue from the top left to the bottom right of each comparison

To investigate the relatedness of WHsTBRF spirochete plasmids to each other, we performed dot plot analysis of plasmid sequences in a pairwise fashion for all species. We were primarily interested in the plasmids that have remained largely syntenic across the WHsTBRF clade. Representative dot plots showing plasmid sequence synteny over a wide genetic distance is shown in Fig. 5. Dot plot analysis highlighted three plasmid families as conserved and largely syntenic across a wide evolutionary distance: the megaplasmid (F6), the small F27 plasmids, and the F28 (cp26-like plasmid). We reasoned that since these plasmids were syntenic they would harbor the majority if not all the non-chromosomal core genes. Indeed, we found that of the 50 non-chromosomal core genes, 46 of these were on the three syntenic plasmid families (Additional File 2: Table S5). Taken together, our analysis of the plasmids indicated that the WHsTBRF have a diverse plasmid repertoire that differs in PF32 and PF57/62-types compared to LD spirochetes and *B. miyamotoi* CT13-2396.

### Comparative genomics of *B. venezuelensis* and *B. turicatae*

The first isolate of *B. venezuelensis* was established in 2018 and a multi-locus sequence analysis placed the species in a monophyletic clade with *B. turicatae*. This relationship indicated a high level of sequence identity between species that were isolated from two different *Ornithodoros* species [15]. We initially predicted that the multi-locus sequence analysis scheme, which used *rrs*, *flaB*, and *glpQ*, was insufficient to resolve *B. venezuelensis* from *B. turicatae* [15]. However, both ANI (Additional File 2: Table S2) and phylogenomic analyses (Fig. 2) maintained this same relationship. The ANI results suggested that *B. venezuelensis* RMA01 and *B. turicatae* may be the same species since they are within the species cutoff of > 95% ANI (~ 98.5%) [37]. However, ANI only analyzes orthologous sequences between the two genomes being compared. If unrelated plasmids are present between the genomes being compared these sequences would not be considered during ANI analysis.

We investigated the plasmids of *B. turicatae* and *B. venezuelensis* RMA01 more closely to better understand the global genomic differences between the two species. We examined the plasmid families represented in both *B. turicatae* isolates and the *B. venezuelensis* RMA01 genomes (Additional File 2: Table S6). Twenty one plasmid families were represented with only four shared between all three genomes (F6, F20, F27, and F28). Only six plasmid families were shared between the two *B. turicatae* isolates and each isolate separately shared a plasmid family with *B. venezuelensis* RMA01. Between all three genomes, *B. turicatae* BTE5EL contained the greatest amount of unique plasmid families (five), whereas *B. turicatae* 91E135 and *B. venezuelensis* RMA01 each contained two unique plasmid families. In terms of the PF57/62-only plasmids, *B. turicatae* 91E135 contained two and both *B. turicatae* BTE5EL and *B. venezuelensis* RMA01 each contained one. These data highlight the variability in plasmid composition even between highly related isolates of the same species (*B. turicatae* 91E135 and BTE5EL).

We also investigated the similarity of *B. venezuelensis* and *B. turicatae* plasmid sequences using dot plot analysis. Both the ANI analysis and dot plots suggested that of the two isolates of *B. turicatae*, *B. venezuelensis* RMA01 was only modestly more related to *B. turicatae* 91E135 (Additional File 2: Table S2, Additional File 1: Fig S9). The similarity was due to the greater synteny of the lp17-2 plasmid of *B. venezuelensis* RMA01 with the lp19 plasmid in *B. turicatae* 91E135 as opposed to the lp46 plasmid in *B. turicatae* BTE5EL. However, outside of the core plasmid families (F6, F27, F28) there was little synteny between *B. turicatae* isolates and the *B. venezuelensis* RMA01 genome.

**Fig. 6:**
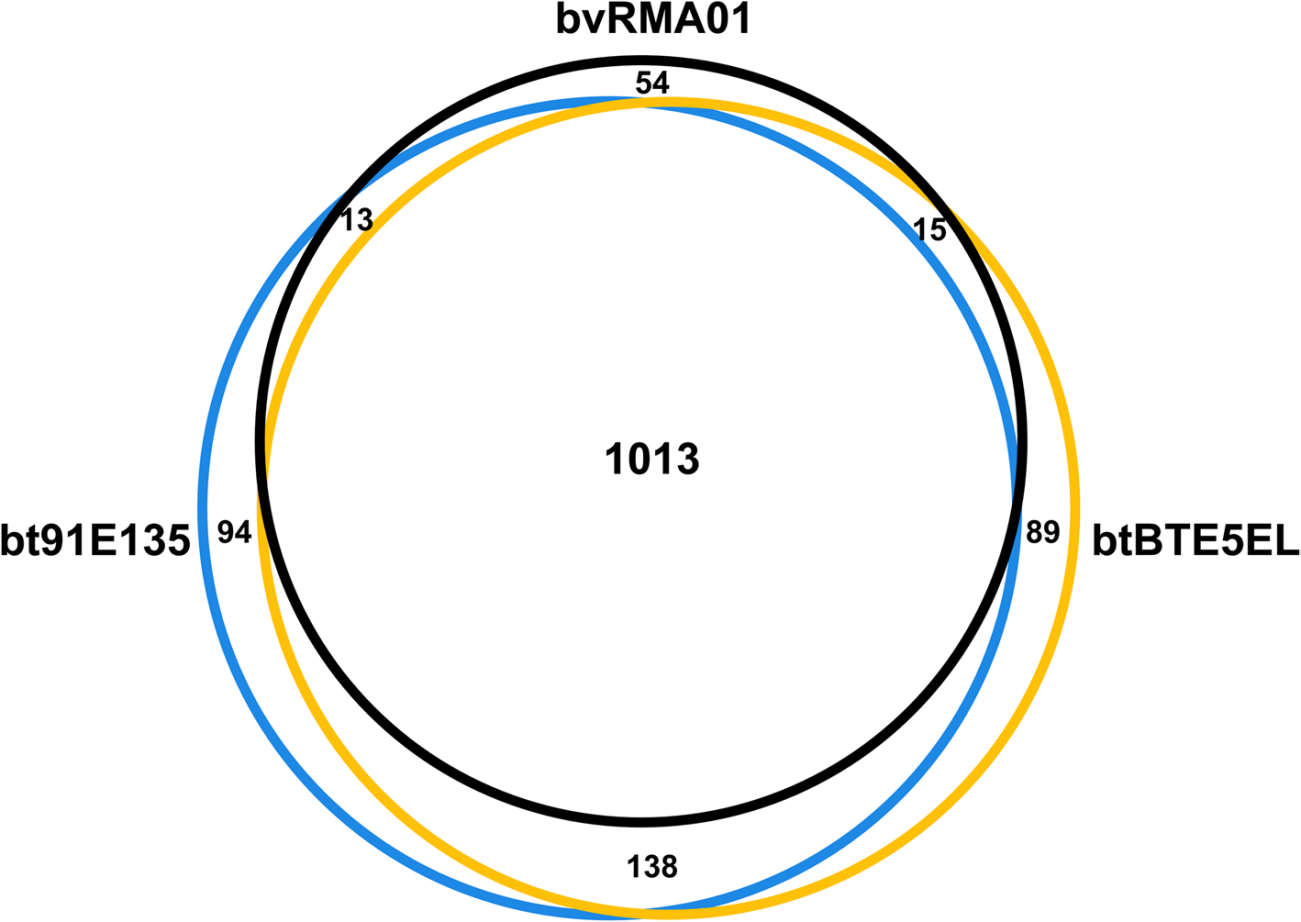
Gene content comparison between *B. turicatae* isolates and *B. venezuelensis* RMA01. To determine the number of gene clusters shared between *B. venezuelensis* RMA01 and the *B. turicatae* isolates an area-proportional Euler diagram was generated. The gene content of the different isolates is represented by different colored circles: blue, bt91E135 = *B. turicatae* 91E135; black, bvRMA01 = *B. venezuelensis* RMA01; yellow, btBTE5EL = *B. turicatae* BTE5EL. The numbers in each region indicate the number of gene clusters present in that relationship

Given our observations, we analyzed the presence and absence output from Panaroo to understand gene content differences between *B. turicatae* and *B. venezuelensis* RMA01. Gene cluster analysis of the chromosomes revealed that there were only four differences between both *B. turicatae* isolates and *B. venezuelensis* RMA01 (*dusA*; group_368_hypothetical protein; group_563_hypothetical protein; and group_95_MATE family efflux transport, Additional File 2: Table S3). Analyzing the plasmid gene clusters, we determined that *B. venezuelensis* RMA01 shared 1,013 gene clusters with both *B. turicatae* isolates but contained 54 unique gene clusters (Fig. 6). We further investigated the gene clusters that were unique to *B. venezuelensis* RMA01 (54 gene clusters) and both *B. turicatae* isolates (138 gene clusters) (Additional File 2: Table S7). Most differentially present gene clusters were annotated as either *hypothetical proteins*, proteins with domains of unknown function, *vmp* alleles, or plasmid partitioning genes. Differences in *vmp* alleles were expected, given the wide variation in sequence between alleles even within the same genome [53, 54], which should result in numerous gene clusters. The difference in plasmid partitioning genes was not surprising given the variation in plasmid content between the two species. Phage-related gene clusters (*mlp-family lipoprotein*, *Borrelia repeat proteins*, tail fiber genes, etc.) were also differentially present between the two species. Moreover, gene cluster differences also reflected structural differences found between the two species. For example, gene clusters functionally annotated as “Borrelial persistence in ticks protein A” genes are located in a region of the megaplasmid that is truncated in *B. venezuelensis* RMA01 and the number of these genes differ between *B. turicatae* (5 copies) and *B. venezuelensis* RMA01 (2 copies). Collectively, these data demonstrate that there is extensive plasmid sequence differences between *B. turicatae* and *B. venezuelensis* RMA01 due to variation in plasmid composition and gene content.

## Discussion

This study generated complete chromosome and plasmid genomes from representative isolates of seven soft TBRF spirochete species found in the Western Hemisphere. With these genomes, we performed a detailed comparative genomics analysis on this RF spirochete clade. We observed plasmid diversity as they clustered into 30 families based on PF32 loci. Interestingly, only three plasmid families were syntenic between all species analyzed. Our findings supported prior observations that *Borrelia* species genomes are in flux with recombination occurring in plasmids and the telomeric ends of the chromosome [20–22, 48, 55–57]. Furthermore, our phylogenomic analysis identified underlying gene tree discordance.

We performed CF analysis on the concatenation-based phylogenomic tree because previous analyses have indicated unstable branches across the WHsTBRF taxa [15, 58–63]. Specifically, studies using the *Borrelia* multi-locus sequence typing (MLST) scheme put forth by Margos et al. are incongruent with phylogenomic analyses of the *Borrelia* species [40, 41, 58–61, 63, 64]. The current *Borrelia* MLST scheme uses eight conserved, chromosomal housekeeping genes (*clpA, clpX, nifS, pepX, pyrG, recG, rplB,* and *uvrA*) [58]. This approach was initially developed for LD spirochetes and later implemented with RF borreliae [59–61, 63]. However, when this scheme has been used with RF spirochetes a branch is formed including *B. hermsii* and *B. anserina* to the exclusion of the rest of the WHsTBRF clade, while *B. miyamotoi* often occurs as a sister clade to the WHsTBRF [59, 61, 63]. There was even one case where MLST phylogenetic analysis placed *B. coriaceae* outside of the WHsTBRF and *B. miyamotoi* clade [60]. In our saturation analysis of the single-copy core gene dataset, *clpX* and *rplB* were identified as having a high risk of saturation indicating that they have lost their phylogenetic signal. Importantly, previously published phylogenomic results contrasted with this MLST analysis [40, 41, 64]. They resolved *B. anserina* and *B. hermsii* into separate branches that included *B. coriaceae* in the WHsTBRF clade and placed the WHsTBRF and Eastern Hemisphere sTBRF spirochetes as sister clades to the exclusion of *B. miyamotoi* [40, 41, 64]. While we did not address the relationship of *B. miyamotoi* to the WHsTBRF clade here, we investigated the underlying gene tree variance in our WHsTBRF spirochete dataset using CF analysis. This showed low CF support for the *B. hermsii*, *B. coriaceae*, and *B. parkeri* branches. Low gCF and sCF scores are typically indicative of discordance within the individual gene trees [65]. This indicates that individual gene trees may be incongruent with the species trees and that phylogenetic analysis of one or a few loci can yield conflicting tree topologies against the species tree, as noted with the MLST phylogenetic data. Thus, one should use caution when trying to apply the *clpA, clpX, nifS, pepX, pyrG, recG, rplB,* and *uvrA* MLST scheme to the WHsTBRF taxa for phylogenetic analysis. To validate our concatenation-based tree, we also performed a coalescence-based tree inference to orthologously estimate a species tree.

The coalescence-based ASTRAL method inferred a species tree that recapitulated the concatenation-based tree topology and indicated discordance for the *B. hermsii*, *B. coriaceae*, and *B. parkeri* branches. ASTRAL is a gene tree summation method that estimates a species tree despite gene tree discordance due to incomplete lineage sorting and the presence of horizontal gene transfer [43, 66]. The quartet data for all branches of the ASTRAL tree had alternative topology quartet frequencies that were approximately equal (q2 = q3) and had a normalized quartet frequency of ~ 89.1%. These data suggested a low-level of incomplete lineage sorting had occurred, which may explain some of the observed gene tree discordance [43, 67]. Additionally, the *B. coriaceae* Co53 branch of the ASTRAL tree had a statistically significant difference in alternative frequencies indicating that incomplete lineage sorting alone could not explain this branch [67]. Sources of gene tree discordance include biological (e.g. horizontal gene transfer) and technical (gene tree estimation error and hidden paralogs) [68, 69]. Given that previous phylogenomic analyses that used different methodologies were congruent with our analysis, we suspect the gene tree discordance seen in our study was biological. Clearly, more work is needed to generate a complete and confident understanding of the evolutionary history of this WHsTBRF spirochete clade.

A pangenome analysis investigated gene diversity across the WHsTBRF clade and was reflective of the numerous paralogous families in the *Borreliaceae*. The WHsTBRF spirochete pangenome consisted of a total of 2,576 unique gene clusters. Previous pangenome analysis by Elbir et al. [28] on African RF borreliae (*Borrelia duttonii*, *Borrelia recurrentis*, *Borrelia crocidurae*, and *Borrelia hispanica*) estimated a pangenome size similar to ours at 2,514 gene clusters. Pangenome analysis by Mongodin et al. [70] estimated pangenome sizes of ~ 1,500 and ~ 1,859 for *B. burgdorferi *sensu stricto and *B. burgdorferi *sensu lato*,* respectively. However, the *Borreliaceae* contain approximately 175 paralogous gene families with intraspecific nucleotide identities often ≥ 60% [20, 27, 71]. Due to the low similarity of these paralogous multicopy gene families within species and between species, gene clustering algorithms will generate multiple clusters for these families. This phenomenon will inflate the overall number of gene clusters reported by pangenome analysis. Indeed, an investigation into the Panaroo output showed that the *variable small protein* (*vsp*) and *variable large protein* (*vlp*) alleles contributed 78 and 282 gene clusters, respectively. This accounted for ~ 14% of the entire pangenome. This caveat should be considered when analyzing pangenome data from *Borrelia* species.

Given that prior work with *B. turicatae* and *B. hermsii* indicated that the chromosomes were largely collinear and had similar gene content, a chromosomal analysis was conducted across the WHsTBRF spirochete clade [28]. Mauve alignment analysis indicated that the chromosomes of the WHsTBRF spirochetes were collinear with no large, internal structural changes. Since we produced full-length chromosomes, the Mauve analysis allowed us to detect variable gene content located at the telomeres. Variable gene content in the 3’ end of LD spirochete chromosomes has been previously reported [21, 57]. In contrast, both the 5’ and 3’ chromosome ends in WHsTBRF spirochetes were not syntenic across all species. For most of the isolates we examined, the gene content on the chromosome ends was also shared with plasmid telomeric sequence.

We investigated plasmid diversity to further appreciate the genomic complexity of RF spirochetes compared to the more characterized LD spirochete genomes. The average number of plasmids found in the WHsTBRF clade (13.9) was higher than that reported for *B. burgdorferi *sensu lato (12.8) but lower than that of *B. burgdorferi *sensu stricto (16.9) [22, 55]. The ratio of circular to linear plasmids for WHsTBRF spirochetes was 0.35 (range: 0–0.83), which is low compared to the range found in LD spirochetes [22, 55]. On average, the circular to linear plasmid ratio of *B. burgdorferi *sensu lato was 0.68 and 0.92 for *B. burgdorferi *sensu stricto [22]. The presence and sizes of circular plasmids were variable between species and even within species.

Our phylogenetic analysis of the 128 plasmids generated in this study identified 30 plasmid families, which are similar in classification to the LD spirochete plasmid-types [22]. Currently there are 30 recognized plasmid types in LD spirochetes based on the PF32 gene [22]. Our analysis found that only 11 RF plasmid families had a related LD spirochete plasmid type. This indicates that there are PF32 types unique to both RF and LD spirochetes based on this classification scheme. We found that, in agreement with previous work done in LD spirochetes and *B. miyamotoi* [20, 22, 25, 50, 55], no genome (with noted exceptions) contained intact PF32 loci of the same family on different plasmids. We identified 13 plasmids that lacked a PF32 locus and only had the PF57/62 locus. Plasmids lacking the PF32 gene have been observed in LD spirochetes (e.g. lp5 and cp9 plasmid-types) [22]. These LD plasmid-types did not phylogenetically cluster with the RF spirochete PF57/62-only loci. This further suggests unique plasmid types in both the WHsTBRF and LD spirochetes. Based on the gene content of the WHsTBRF PF57/62-only plasmids, we hypothesize that they are either remnants of or intact *Borrelia* prophages.

Deeper analysis of both circular and linear plasmids indicated the presence or remnants of prophage genomes. We identified prophage associated genes including *blyA* and *blyB* holin genes, *mlp family lipoproteins*, and phage recombinase genes. Variation in phage associated plasmids between the strains and species evaluated suggested differences in phage exposure. This variation is noted in *B. parkeri* SLO. This isolate contained multiple circular plasmids encoding phage-related genes. Interestingly, prior work with other *B. parkeri* strains (which did not include the SLO strain) found only linear plasmids [34]. The geographic location, reservoir host use, and variations between tick populations could drive differences in phage-related plasmid composition through different phage exposures [72–74]. Prophages have been documented in LD spirochetes [72, 74, 75], and these viruses have been isolated and used to mediate transduction between *B. burgdorferi* isolates [74, 76, 77]. Future work should investigate whether there are inducible phages in RF spirochete species and whether these viruses can mediate transduction or horizontal gene transfer. This would provide a mechanism for genome plasticity of RF spirochetes.

We concluded our analyses by comparing the *B. venezuelensis* RMA01 genome to that of *B. turicatae* 91E135 and BTE5EL. The genomes of *B. venezuelensis* RMA01 and *B. turicatae* 91E135 and BTE5EL were within the species cutoff for prokaryotes (> 95%) [37]. Interestingly, previous reports for borrelial genomes have identified species with similarly high ANI scores. Elbir et al. [28] and Adeolu and Gupta [78] reported ANI scores between *Borrelia* (*Borreliella*) *garinii* and *Borrelia* (*Borreliella*) *bavariensis* (~ 98%), *B. recurrentis* and *B. duttonii* (~ 99%), *B. duttonii* and *B. crocidurae* (~ 99%), and *B. crocidurae* and *B. recurrentis* (~ 99%). While *B. crocidurae*, *B. duttonii*, and *B. recurrentis* had high pairwise ANIs, these species utilize different tick or insect vectors for their life cycle [3, 79–83]. ANI does not consider differences in plasmid composition between species.

A biological feature of RF spirochetes that has been used to speciate the pathogens is vector specificity, which should be considered with *B. venezuelensis* and *B. turicatae*. With vector specificity, a given spirochete species colonizes and is transmitted by a given species of argasid tick [84–87]. We report here that there is a high level of sequence identity between *B. turicatae* and *B. venezuelensis* RMA01 despite being isolated from two different tick *Ornithodoros* species whose known distributions are separated by at least 2,300 km [88, 89]. However, prior to considering *B. turicatae* and *B. venezuelensis* as the same species, vector competence studies are needed to determine whether *Ornithodoros rudis* (the vector of *B. venezuelensis*) can maintain and subsequently transmit *B. turicatae.* Similarly, studies would be needed with *O. turicata* (the vector of *B. turicatae*) and *B. venezuelensis.* Collectively, these findings would better define the biological and ecological differences between *B. venezuelensis* and *B. turicatae*.

Limitations of this work include the small number of species in this clade and shortcomings with the software used in the analyses. RF has been reported in Mexico [90–92], Central America [93–95], and throughout South America [96–99], yet we only had two representative species from Latin America [9, 15]. Furthermore, the genome assembly software used was not designed necessarily for covalently closed linear replicons [100]. As a result, borrelial genome assembly is labor intensive and can result in errors [24, 101, 102]. We mitigated assembly errors by implementing quality assessment steps utilized by Kuleshov and co-workers [25], and incorporated a new tool, Merqury [103]. Annotation was also complicated by the lack of characterization for many borrelial proteins resulting in many genes being classified as “hypothetical proteins” or “domain of unknown function-containing proteins”. Another annotation/gene classification shortcoming was found in the classification of pseudogenes (e.g*. B. hermsii* lp73 and lp89, *bhYOR_001322* and *bhYOR_001456*) and subsequently when analyzing the InterProScan data for pseudogenes. There were instances where InterProScan analysis failed to classify plasmid partitioning pseudogenes that the annotation software had classified. Lastly, the number and diversity of the borrelial paralogs complicates proper estimation of the core and accessory genomes. Regardless, the completed chromosome and plasmid genomes enabled an informative comparative genomics analysis on the WHsTBRF clade.

TBRF spirochetes are a globally distributed yet neglected disease, and the genomes generated by this study will provide needed resources to study pathogenesis, vector biology, and for the development of diagnostics. With only two known isolates of TBRF spirochete from Latin America, the genomes of *B. venezuelensis* RMA01 and *B. puertoricensis* n. sp*.* SUM provides the opportunity to commence vector competence studies and to determine the clinical manifestation of *B. venezuelensis.* In Brazil, a Lyme-like illness (Baggio-Yoshinari Syndrome) circulates in the country but there is no epidemiological or ecological support for *B. burgdorferi* [104, 105]. Similarly, in the southwestern United States *B. turicatae* can present with neurological symptoms similar to Lyme disease and is often misdiagnosed [14, 106]. Studies are now possible to ascertain the pathogenesis of *B. venezuelensis* and to determine whether it is phenotypically like *B. turicatae.* Furthermore, there are likely more species to be found throughout the entirety of the RF *Borrelia* clade, some of which may be important species in the evolutionary history of this group. As *Borrelia* spp. isolation becomes more feasible [107], efforts are needed to gain a more complete understanding of their genome evolution, plasmid diversification, and how this may impact pathogenesis and vector-host use.

## Conclusions

Given the unique genomic structure of spirochetes in the *Borreliaceae* family and the paucity of completed chromosome and plasmid genomes for RF spirochetes, this work addressed important knowledge gaps and provides needed resources. This work tripled the number of complete, plasmid-resolved genomes for soft tick-borne RF *Borrelia* species found in the Western Hemisphere and we provided a comparative genomics analysis. We investigated outstanding questions in the field regarding intra- and inter-species plasmid relationships and the high degree of sequence identity between *B. turicatae* and *B. venezuelensis,* which were isolated from two different *Ornithodoros* species. From these analyses we have identified substantial diversity in the plasmid sequences between the WHsTBRF spirochetes, as well as a core group of plasmids found in all isolates analyzed. Furthermore, while *B. turicatae* and *B. venezuelensis* RMA01 are highly similar at the chromosome-level, at the plasmid-level there were substantial differences in composition, structure, and gene content. Collectively, our findings will be foundational for future endeavors to identify the plasmid genetic elements that play a role in vector and host adaptation for both relapsing fever spirochetes and the *Borreliaceae* as a whole.

## Methods

### Borrelial strains and culturing

*Borrelia* spp. and isolates used in this study are referenced in Table 1. The seven species we selected are the only soft tick-borne relapsing fever *Borrelia* species isolated in the Western Hemisphere. The particular isolates were chosen because they are either the only isolates available of that species (*B. puertoricensis* n. sp. SUM and *B. venezuelensis* RMA01), conventional lab strains (*B. hermsii* DAH, *B. hermsii* YOR, *B. coriaceae* Co53, *B. parkeri* SLO and *B. turicatae* 91E135), or are from a species with limited isolates available (*B. anserina* BA2). The *B. turicatae* BTE5EL was also included because 1) it is the only human isolate and 2) from our previous work analyzing *B. turicatae* isolates, BTE5EL and 91E135 are from different *rrs*-*rrlA* intergenic spacer genotypes. All of the isolates were low passage (< 10), polyclonal isolates grown in either mBSK with 12% heat-inactivated rabbit serum, at either 35° or 37 °C in a 5% CO_2_ environment or BSK-R (*B. puertoricensis* n. sp*.* SUM and *B. venezuelensis* RMA01) at 34° C with caps sealed in a microaerobic environment [107]. Initial cultures were grown in 4 mL of media in sealed 8 mL polystyrene tubes (*B. puertoricensis* n. sp*.* SUM and *B. venezuelensis* RMA01 were cultured in 4.5 mL of media in sealed 5 mL polystyrene tubes) to ~ 10^7^ cells/mL and were then used to inoculate 45 mL of media in a 50 mL polypropylene tube and cultured until ~ 10^7^ cells/mL.

### Genomic DNA isolation and sample quality control

Genomic DNA was extracted from the 45 mL of culture (see above) using phenol–chloroform, as previously described [108]. The resulting DNA pellet was allowed to air dry and was resuspended in 1 × TE buffer and stored at 4 °C. After ~ 48 h at 4 °C the genomic DNA was quantified using a NanoDrop 2000 (Thermo Fisher Scientific, USA). Genomic DNA samples were assessed for quality using pulsed-field gel electrophoresis to determine the integrity of the chromosome and plasmids, as previously described [33].

### Sequencing

#### Oxford nanopore technologies library preparation

All strains were sequenced on an Oxford Nanopore Technologies (ONT) MinION Mk1B sequencer using the R9.4.1 flow cell and the SQK-RBK004 rapid barcoding library preparation kit. Four different library preparation strategies were attempted to yield larger read sizes while decreasing the amount of free adapter sequence. Initial library preps (library prep# 1) were done without prior cleanup of genomic DNA however we found that any genomic DNA that did not have an A260/280 or A260/230 ≥ 1.8 yielded little sequencing data. This result was likely due to interference of salts and/or organics in the transposase barcoding reaction. Therefore, in subsequent library preps (library prep# 2 and 3) all genomic DNA was further purified using one volume of magnetic beads (Omega Biotek, USA), washed twice with 70% ethanol, and eluted in pre-warmed (56 °C) elution buffer (Buffer EB; Qiagen, Germany). The specific library preparation strategies utilized for each strain is indicated in Additional File 2: Table S8.

#### Illumina sequencing

Bead-purified genomic DNA (discussed above) was provided for each strain to the Microbial Genome Sequencing Center (MiGS Center, USA) for Illumina sequencing. Sequencing performed using the Illumina Nextera paired-end 2 × 150 bp library prep kit on a NextSeq 550 sequencer. Demultiplexed FASTQ files were provided by the MiGS Center.

### Sequence analysis

Example commands for each software are given in Additional File 5.

#### Sequence data processing

ONT data was basecalled using ONT’s Guppy v3.6.0 or 4.0.14 yielding FASTQ files of raw, basecalled data. The raw, basecalled data was demultiplexed using guppy_barcoder with the “–detect_mid_strand_barcodes” option. The FASTQ files after demultiplexing were initially processed using NanoFilt (v2.7.0) to remove all reads less than 1 kb and quality scores less than seven [109]. Summary statistics for each isolate’s ONT data were generated using NanoStat (v1.2.0) (Additional File 2: Table S9) [109]. Illumina data was provided by the MiGS Center as FASTQ files and filtered using Fastp (v0.20.1) to remove reads below Q20, trim adapters, and perform read correction (Additional File 2: Table S9) [110]. The Illumina data was merged for short-read polishing but was also processed as separate reads which were used for the Merqury tool (v1.1) for quality assessment and genome refinement (discussed later) [103]. Sequencing read data were deposited in NCBI’s Sequence Read Archive (SRA) as FASTQ files for both ONT and Illumina datasets. These data were deposited under the BioSample accession number associated with each isolate (see Additional File 2: Table S1).

#### Assembly and polishing

Whole genome assemblies were generated following the bioinformatics pipeline developed by Kuleshov et al. [25]. In short, filtered ONT data was assembled with the Canu (v2.0) assembler [100]. Following assembly, circular contigs were trimmed by using the apc.pl script to remove overlapping ends [111]. Contigs were then assessed for redundancy using BLASTn (v2.11.0) and by self-vs-self dot plot assessment using FlexiDot (v1.06) [112, 113], and redundant contigs were removed. Inverted telomeric ends of linear replicons were trimmed using Unipro UGENE (v35) for visualization and sequence manipulation, and the online EMBOSS einverted tool (http://emboss.toulouse.inra.fr/cgi-bin/emboss/einverted) was used to identify the hairpin sequence [114]. After inverted telomeric ends were removed, assemblies were polished using both ONT and Illumina data. ONT polishing was done using three iterative rounds of Racon (v1.4.16) polishing and one round of Medaka (v1.0.3) polishing using the g3.6.0 model [115, 116]. The ONT polished assembly was further polished with the merged Illumina data using iterative rounds of Pilon (v1.23) using the “all” option [117]. Pilon was run iteratively until no changes were made, typically two to four rounds though *B. coriaceae* Co53 required eight iterations.

#### Genome assembly quality control and refinement

Final genome assemblies fit the following quality criteria: 1) linear replicons must be from telomere to telomere, circular replicons must have no telomeres and be contiguous from end to end; 2) all replicons must be complete (i.e. 1 replicon = 1 contig); and 3) all assemblies must have a ≥ Q40 Phred-quality score and a ≥ 99.5% completeness score as calculated by Merqury [103].

Assemblies were initially assessed by Quast (v5.0.2) for mapping percentage of the Illumina and ONT datasets [118]. Coverage was determined for each of the replicons via Mosdepth (v0.2.9), and mapping via Minimap2 (v2.17-r974) with visualization in Geneious (v2020.1.2; Biomatters, New Zealand) [119, 120]. Each replicon’s sequencing coverage and depth was calculated to ensure there were no plasmid fusions or assembly errors. However, read mapping as a quality metric of completeness can be misleading. Currently there are no high-quality, complete reference genomes for many of the species we investigated which could be used to determine the completeness and quality of our assemblies. Thus, we employed the reference-free quality assessment tool Merqury. Merqury utilizes unmerged paired-end Illumina data to assess assembly completeness as well as quality at the individual contig and whole-assembly levels. Completeness is determined by how much of the unique k-mer fraction is contained in the assembly of interest; subsequently, quality is determined by measuring the amount of unique k-mer identity clashes in the assembly.

Merqury was also used for assembly refinement in situations where completeness was shown to be low (< 99.5%). Illumina reads containing unique k-mers not found in the assembly were extracted. After extracting these reads, they were assembled into scaffolds using the SPAdes assembler (v3.13.1). These scaffolds were used as a reference to map the ONT read dataset for that isolate using Minimap2 [121]. The resulting BAM file was visualized with Geneious and reads were assessed to find plasmids that may not have been assembled in the initial assembly. Often, missing plasmids were very similar to a plasmid already in the assembly, which was typical for many of the circular plasmids. We observed that often assemblies that were missing plasmids had to undergo greater than three Pilon iterations until no changes were made. After adding the missing plasmid(s), the number of Pilon iterations required usually decreased whereas completeness and quality, as assessed by Merqury, increased. Assemblies were generally considered refined, complete, and of high-quality if there was no plasmid that could be readily identified from the SPAdes scaffolds of unassembled unique k-mer reads, the completeness score was ≥ 99.5%, and had an assembly quality score ≥ Q40.

#### Genome annotation, InterProScan analysis, and replicon orientation

The plasmids of the final assembly were named based on topology (lp = linear plasmid, cp = circular plasmid) and size (nearest kilobase). In instances where the size was the same between two plasmids the plasmid was further numbered such as lp28-1, lp28-2, etc. The assemblies post-Merqury were annotated via a local copy of NCBI’s prokaryotic genome annotation pipeline (PGAP, v2020-09–24.build4894) to identify and annotate all coding sequences and genome features and InterProScan supplemented this with protein classification [122, 123]. The resulting GFF file was used to extract the transcripts from the assembly FASTA file using GFFread (v0.12.2) [124]. The extracted transcripts were used as input to a local copy of InterProScan (v5.47–82.0) to further classify transcripts using the CDD, Pfam, and Superfamily databases [125–127]. To facilitate standardization of replicon orientation, we identified the PF57/62 plasmid partitioning gene that was intact and had the best hit from InterProScan (Pfam, PF02414) on each replicon and oriented all plasmid replicons to put this gene in the positive-sense. The megaplasmid was kept with the PF57/62 gene in the negative-sense because previous studies and analyses have been conducted with the PF57/62 gene in the negative-sense [128, 129]. The chromosome only had PF32 plasmid partitioning genes where three were put in the positive-sense which put the remaining gene in the negative-sense, this serendipitously matched previously published chromosomal sequence orientations. Circular plasmids were reoriented to start with the best hit PF57/62 gene oriented in the positive-sense. Re-oriented genomes were then annotated with PGAP and analyzed via InterProScan as before. All subsequent work was completed using the final, re-oriented genomes.

#### Average nucleotide identity

Average nucleotide identity was determined by FastANI (v1.32) using an all-versus-all approach on the genome and chromosome FASTA files for all the strains being investigated [37]. The –matrix option was used to generate an identity matrix.

#### Pangenome analysis

Pangenome analysis was carried out using the Panaroo pipeline (v1.2.3) [38]. We modified Panaroo parameters to account for particular aspects of our dataset. To account for analyzing multiple species that at the lowest pairwise ANI was ~ 85% we adjusted the –threshold parameter to 0.8. To account for the high number of paralogous gene families we used the –merge_paralogs options. To account for our assemblies’ complete and non-fragmented linear replicons, we modified –min_trailing_support and –trailing_recursive to 0 to prevent erroneous trimming of the replicon ends. All refound pseudogenes (indicated in the output by refound….._stop) were manually removed from all analyses. Visualization of the Panaroo output was done using the roary_plot.py from Roary (v3.13.0) using the Newick tree generated from our concatenation-based phylogenomic tree (see *Phylogenomics and Concordance Factor Analysis*) and the gene_presence_absence_roary.csv file from Panaroo [130]. To visualize the pangenome and core genome line graph, PanGP (v1.0.1) was used with the gene_presence_absence.Rtab file from Panaroo [131]. The gene presence/absence file that lists the gene loci for each isolate in each gene cluster is found in Additional File 6.

#### Phylogenomics and concordance factor analysis

Concatenation-based phylogenomic analysis was performed using the Panaroo pipeline including our WHsTBRF spirochete dataset and the *B. miyamotoi* CT13-2396 genome. From the MAFFT alignments generated by Panaroo of the core genes, we analyzed the 741 single-copy core genes for substitution saturation using PhyloMAd [132]. Genes identified by PhyloMAd as having a high risk of substitution saturation in the phylogenetically informative sites (Risk_Entropyvar) were removed from phylogenomic analysis [39]. The remaining 650 single-copy core genes were used to infer a species tree and concordance factor analysis. See Additional File 3 for a list of the genes used for analysis (Risk_Entropyvar = low.risk) and those not used (Risk_Entropyvar = high.risk). A maximum likelihood tree was inferred with IQ-TREE2 (v2.1.2) using an edge-linked proportional partition model, automatic gene alignment concatenation, and 1,000 ultrafast bootstrap replicates [133–136]. The resulting tree and analysis were used to infer gene trees in IQ-TREE2 followed by gene and site concordance factor calculation. The final tree with the bootstrap and concordance factor metadata was analyzed using FigTree and annotated in Inkscape. Concordance factor analysis and interpretation are discussed in-depth in [65].

Coalescence-based phylogenomic analysis was performed on the 650 single-copy core gene alignments as discussed above. For this analysis however, each individual gene tree was bootstrapped with 1,000 ultra-fast bootstraps and collapsed at branches with < 10% support using IQ-TREE2. The resulting trees were used to infer a coalescence-based tree using ASTRAL [43, 66, 67, 137, 138]. The alternative quartet frequencies were evaluated using a test of proportions (prop.test) in R (v4.0.4) to assess whether the frequencies were statistically unequal. The resulting tree was visualized and annotated as a cladogram in FigTree and Inkscape, respectively.

#### MUMmerplot dot plot analysis

Dot plot analysis of the plasmid sequences and plasmid groups was performed using MUMmer (v4.0.0beta2) [139]. The sequences were first aligned using Nucmer with the –maxmatch option to allow for visualization of repeats and redundant sequence found within and between two sequences. The dot plots were generated using MUMmerplot with default options and the output from Nucmer to generate a PNG file.

#### Plasmid partitioning gene phylogenetic analysis and nucleotide sequence clustering

Plasmid partitioning genes were assessed using the results of each genome’s InterProScan analysis and PGAP’s annot.gff file. This analysis was performed using our WHsTBRF spirochete dataset, and deposited data for the LD spirochete dataset and *B. miyamotoi* CT13-2396 (Additional File 2: Table S4). Plasmid partitioning genes were found using the CDD and Pfam databases (PF32 = cd02038 and cd02042, PF49 = PF01672, PF50 = PF0289, PF57/62 = PF02414 based on work by Kuleshov et al. [25]) and located using the annot.gff file. Plasmid partitioning genes (PF32, PF49, PF50, PF57/62) identified by InterProScan using the above methods are available in Additional File 7. Using the PF32 or PF57/62 loci nucleotide sequences, we performed phylogenetic analysis using maximum likelihood tree inference. The nucleotide sequences of either the PF32 or PF57/62 loci were aligned using MAFFT with the “–auto" option. The alignment was used to generate a maximum likelihood tree inferred by IQ-TREE2 with 1,000 ultrafast bootstraps and the “–polytomy” option. Branches were collapsed with supports less than 50% using the “-minsupnew” option. The tree was analyzed using FigTree and annotated in Inkscape. Both phylogenies were analyzed using FigTree and annotated in Inkscape. The PF32 loci nucleotide sequences used for phylogenetic analysis were clustered using CD-HIT-EST (v4.8.1) with an 85% sequence identity threshold (-c 0.85) [140].

#### Euler diagram analysis

Using the binary output from Panaroo for the presence/absence analysis, we extracted the bvRMA01, bt91E135, and btBTE5EL columns. These data were used to plot a Venn diagram in R (v4.0.4) using gplots package (v3.1.1) and venn function [141]. The data from that diagram was then used to generate an area-proportional Euler diagram using the online eulerr application with the disjoint combinations option selected and a seed of 3 used [142]. The Euler diagram was annotated in Inkscape.

#### Mauve

ProgressiveMauve (v20150226 build 10) was used to visualize sequence similarities for the chromosome [45]. Individual GBK files for each genome’s annotated chromosome was used for alignment for the WHsTBRF spirochete dataset. ProgressiveMauve was run without assuming collinearity, with the “seed families” option selected, but otherwise with default options. Visualization was done using the Mauve program.

## Supplementary Information

**Additional file 1:** **Figure S1-9.**

**Additional file 2:** **Table S1-9.**

**Additional file 3.** PhyloMAd saturation test output.

**Additional file 4.** CD-HIT-EST nucleotide sequence identity cluster output of PF32 gene loci.

**Additional file 5.** Example commands.

**Additional file 6.** Panaroo presence/absence output.

**Additional file 7.** Plasmid Partitioning Gene Loci Interproscan Information.

## Data Availability

Genomic DNA for the isolates used in this study is available upon request with the appropriate Material Transfer Agreement. All datasets generated during the current are a part of the PRJNA637792 NCBI BioProject (https://www.ncbi.nlm.nih.gov/bioproject/PRJNA637792). Individual NCBI BioSample accessions are as follows: *B. anserina* BA2 (SAMN18441552), *B. coriaceae* Co53 (SAMN18441555), *B. hermsii* DAH (SAMN18441553), *B. hersmii* YOR (SAMN18441554), *B. parkeri* SLO (SAMN18441556), *B. puertoricensis* n. sp. SUM (SAMN19000909), *B. turicatae* 91E135 (SAMN18441557), *B. turicatae* BTE5EL (SAMN18441558), and *B. venezuelensis* RMA01 (SAMN15156993). NCBI GenBank accession numbers for each replicon are listed below: *B. anserina* BA2. Chromosome, CP073130. lp10, CP073131. lp12-1, CP073132. lp12-2, CP073133. lp27, CP073134. lp88, CP073135. *B. coriaceae* Co53. Chromosome, CP075076. cp29, CP075077. cp30-1, CP075078. cp30-2, CP075079. cp30-3, CP075080. cp48, CP075081. lp11, CP075082. lp188, CP075083. lp20, CP075084. lp23, CP075085. lp24-1, CP075086. lp24-2, CP075087. lp28-1, CP075088. lp28-2, CP075089. lp29-1, CP075090. lp29-2, CP075091. lp30, CP075092. lp31, CP075093. lp32, CP075094. lp33, CP075095. lp34, CP075096. lp38, CP075097. lp39, CP075098. lp60, CP075099. *B. hermsii* DAH. Chromosome, CP073136. cp30-1, CP073137. cp30-2, CP073138. cp55, CP073139. cp90, CP073140. lp12, CP073141. lp185, CP073142. lp26, CP073143. lp27, CP073144. lp28, CP073145. lp31, CP073146. lp59, CP073147. *B. hermsii* YOR. Chromosome, CP073148. cp30-1, CP073149. cp30-2, CP073150. cp60, CP073151. lp11, CP073152. lp194, CP073153. lp23, CP073154. lp46, CP073155. lp73, CP073156. lp89, CP073157. lp93, CP073158. *B. parkeri* SLO. Chromosome, CP073159. cp28, CP073160. cp29, CP073161. cp58, CP073162. lp10, CP073163. lp157, CP073164. lp18, CP073165. lp24, CP073166. lp28, CP073167. lp30-1, CP073168. lp30-2, CP073169. lp32, CP073170. lp34-1, CP073171. lp34-2, CP073172. lp42, CP073173. lp58, CP073174. lp60, CP073175. *B. puertoricensis* SUM. Chromosome, CP075379. cp27, CP075380. cp28-1, CP075381. cp28-2, CP075382. cp29-1, CP075383. cp29-2, CP075384. cp30, CP075385. cp59-1, CP075386. cp59-2, CP075387. cp60, CP075388. cp61, CP075389. lp10, CP075390. lp110, CP075391. lp25, CP075392. lp29-1, CP075393. lp29-2, CP075394. lp35, CP075395. lp39, CP075396. lp41, CP075397. lp46, CP075398. lp47, CP075399. lp57, CP075400. lp65, CP075401. *B. turicatae* 91E135. Chromosome, CP073176. cp30-1, CP073177. cp30-2, CP073178. cp31, CP073179. cp49, CP073180. lp10, CP073181. lp158, CP073182. lp19, CP073183. lp24, CP073184. lp29, CP073185. lp36, CP073186. lp38, CP073187. lp40, CP073188. lp43, CP073189. lp48, CP073190. lp53, CP073191. *B. turicatae* BTE5EL. Chromosome, CP073192. cp30-1, CP073193. cp30-2, CP073194. cp31, CP073195. cp60, CP073196. lp10, CP073197. lp160, CP073198. lp24, CP073199. lp28, CP073200. lp30, CP073201. lp32, CP073202. lp34, CP073203. lp37, CP073204. lp42, CP073205. lp45, CP073206. lp46, CP073207. lp49, CP073208. *B. venezuelensis* RMA01. Chromosome, CP073220. cp28, CP073221. lp10, CP073222. lp127, CP073223. lp17-1, CP073224. lp17-2, CP073225. lp25, CP073226. lp30, CP073227. lp31, CP073228. lp35, CP073229. lp37, CP073230. The individual read datasets generated by this study are available in the NCBI Sequence Read Archive (SRA) (www.ncbi.nlm.nih.gov/sra/). The Illumina raw read dataset for each isolate is available at the following links: *B. anserina* BA2 (https://www.ncbi.nlm.nih.gov/sra/?term=SRR15006057), *B. coriaceae* Co53 (https://www.ncbi.nlm.nih.gov/sra/?term=SRR15006046), *B. hermsii* DAH (https://www.ncbi.nlm.nih.gov/sra/?term=SRR15006056), *B. hermsii* YOR (https://www.ncbi.nlm.nih.gov/sra/?term=SRR15006047), *B. parkeri* SLO (https://www.ncbi.nlm.nih.gov/sra/?term=SRR15006044), *B. puertoricensis* n. sp. SUM (https://www.ncbi.nlm.nih.gov/sra/?term=SRR15006045), *B. turicatae* 91E135 (https://www.ncbi.nlm.nih.gov/sra/?term=SRR15006042), *B. turicatae* BTE5EL (https://www.ncbi.nlm.nih.gov/sra/?term=SRR15006041)., *B. venezuelensis* RMA01 (https://www.ncbi.nlm.nih.gov/sra/?term=SRR15006043). The Oxford Nanopore Technologies FASTQ raw read dataset for each isolate is available at the following links: *B. anserina* BA2 (https://www.ncbi.nlm.nih.gov/sra/?term=SRR15006040), *B. coriaceae* Co53 (https://www.ncbi.nlm.nih.gov/sra/?term=SRR15006053), *B. hermsii* DAH (https://www.ncbi.nlm.nih.gov/sra/?term=SRR15006055), *B. hermsii* YOR (https://www.ncbi.nlm.nih.gov/sra/?term=SRR15006054), *B. parkeri* SLO (https://www.ncbi.nlm.nih.gov/sra/?term=SRR15006051), *B. puertoricensis* n. sp. SUM (https://www.ncbi.nlm.nih.gov/sra/?term=SRR15006052), *B. turicatae* 91E135 (https://www.ncbi.nlm.nih.gov/sra/?term=SRR15006049), *B. turicatae* BTE5EL (https://www.ncbi.nlm.nih.gov/sra/?term=SRR15006048)., *B. venezuelensis* RMA01 (https://www.ncbi.nlm.nih.gov/sra/?term=SRR15006050).
