# Supplementary material for: Comparative genomics of the Western Hemisphere soft tick-borne relapsing fever borreliae highlights extensive plasmid diversity
Source: BMC Genomics. 2022 May 31;23:410. doi: 10.1186/s12864-022-08523-7 (PMC9158201; doi:10.1186/s12864-022-08523-7)
Supplement: Supplementary file 1 — Additional file 1: Figure S1-9. [file 12864_2022_8523_MOESM1_ESM.docx]

**Additional File 1**

**Figure S1
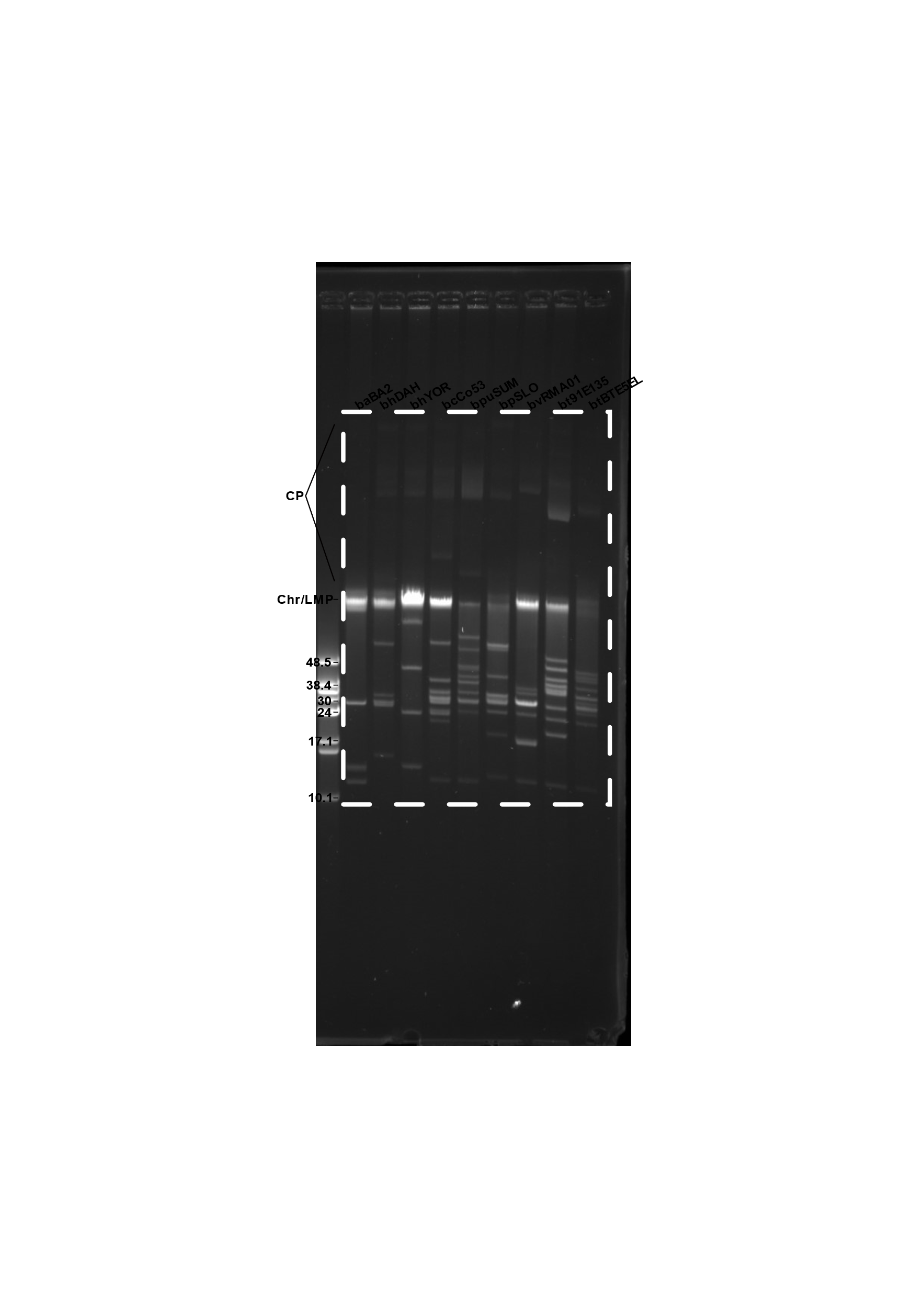
**

**Figure S1.** Full-length pulsed-field gel of WHsTBRF genomic DNA samples. This is the full-length gel image seen in **Figure 1**. Where the image was cropped is indicated by the dashed lines.

**Figure S2**

**
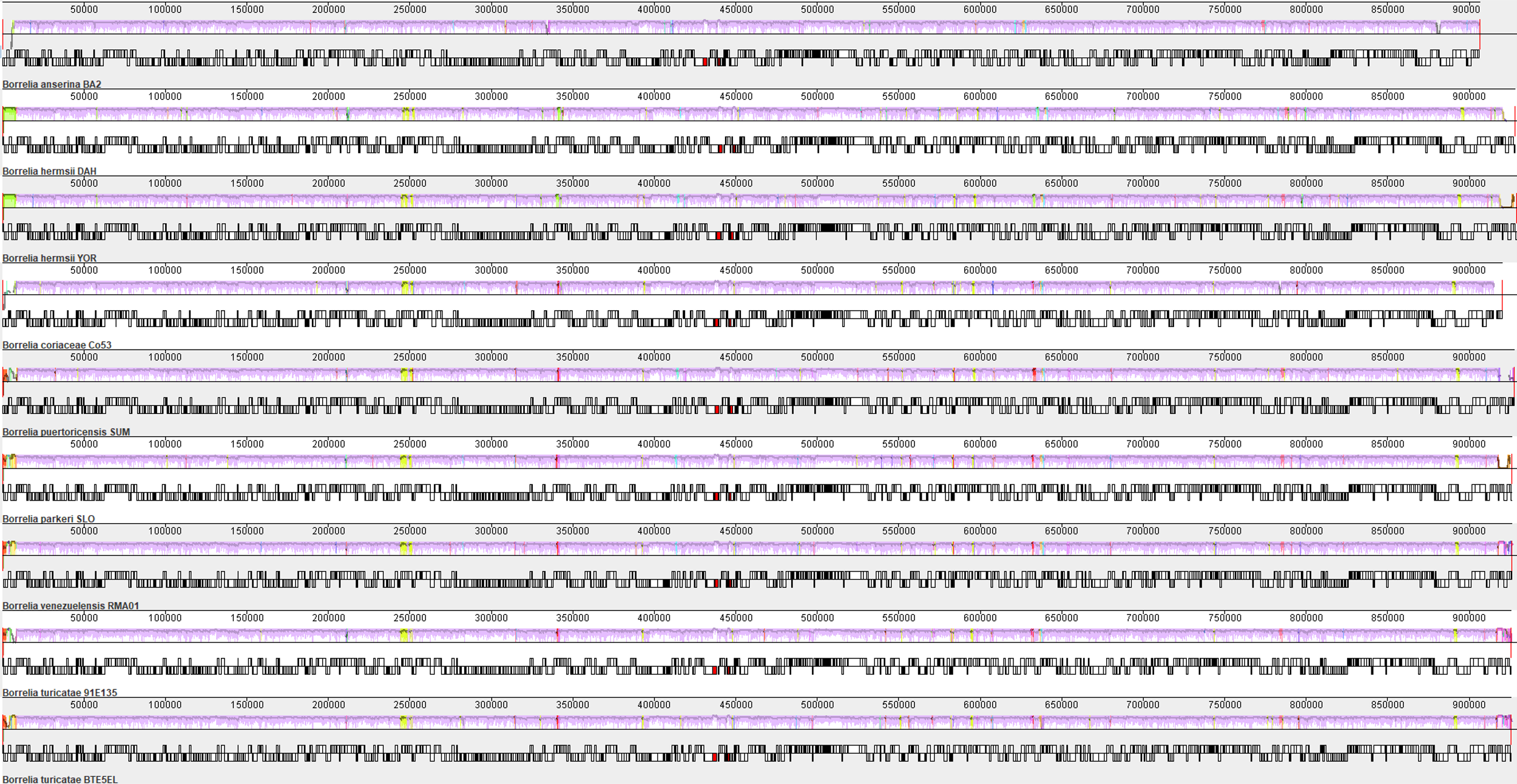
**

**Figure S2.** Mauve alignment of WHsTBRF spirochete chromosome sequences. Chromosome sequences were aligned with Mauve without assuming collinearity and with the “seed families” option. The figure shows the Backbone color scheme with similarity plots since only one collinear block was determined across the isolates. The similarity block colors indicate similar regions between isolates and the colored histograms within the similarity block correspond to nucleic acid similarity (the higher the bar the higher the nucleic acid identity). Genes are shown for each isolate below the similarity box, with genes above the mid-line in the positive-sense and those below in the negative-sense.

**Figure S3**

**
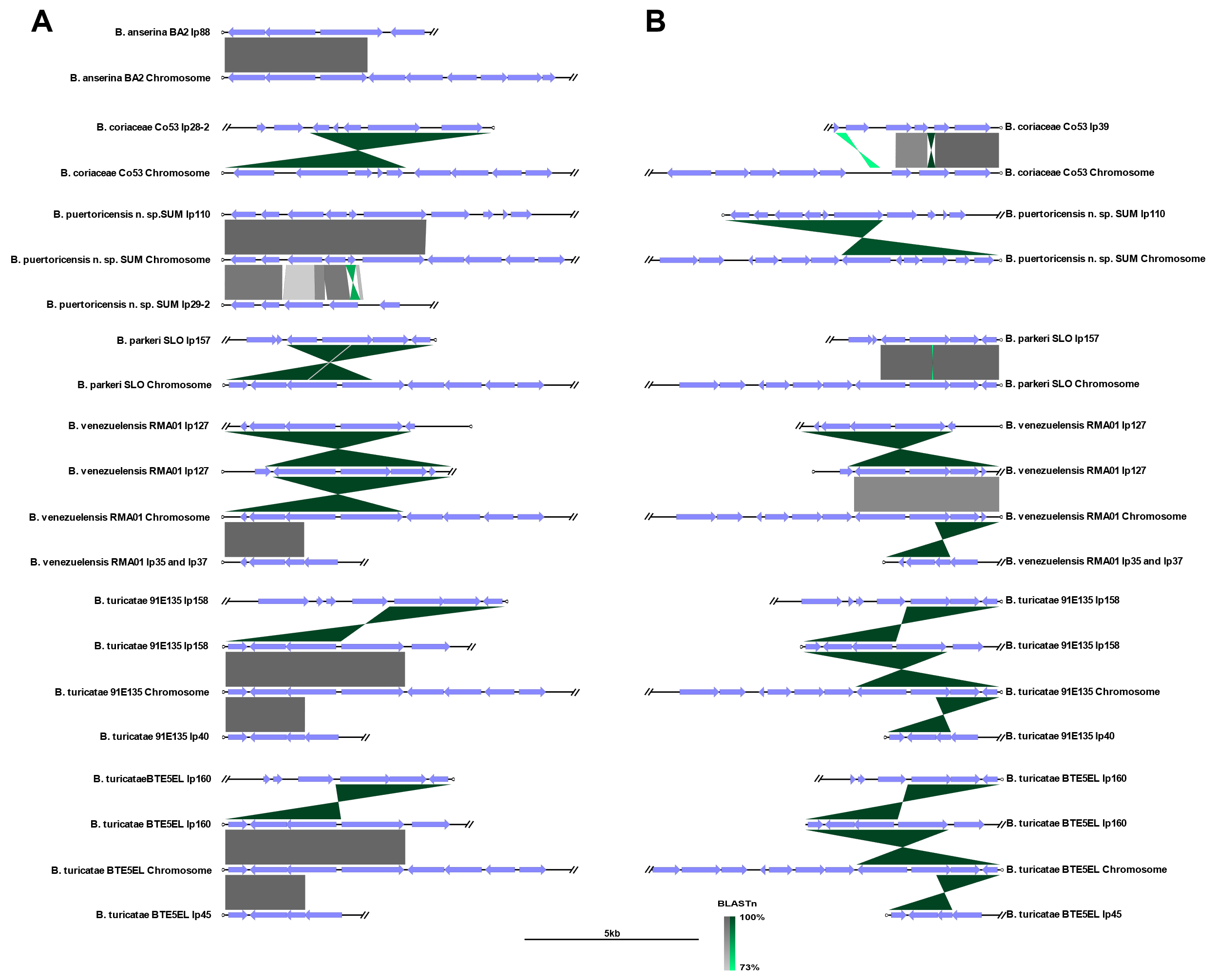
**

**Figure S3**. Visualization of chromosome and plasmid recombination. Plasmid sequences were compared against their respective chromosomes by BLASTn to find regions of similarity between the telomeres using EasyFig. Telomeres are indicated by hairpin loops with breaks in plasmid length for visualization represented by double hashes. ORFs are indicated by purple arrows. BLASTn results scale from 73-100% going from light grey to dark grey whereas inversions scale similarly but from light green to dark green. A scale indicating 5kb in length is shown.


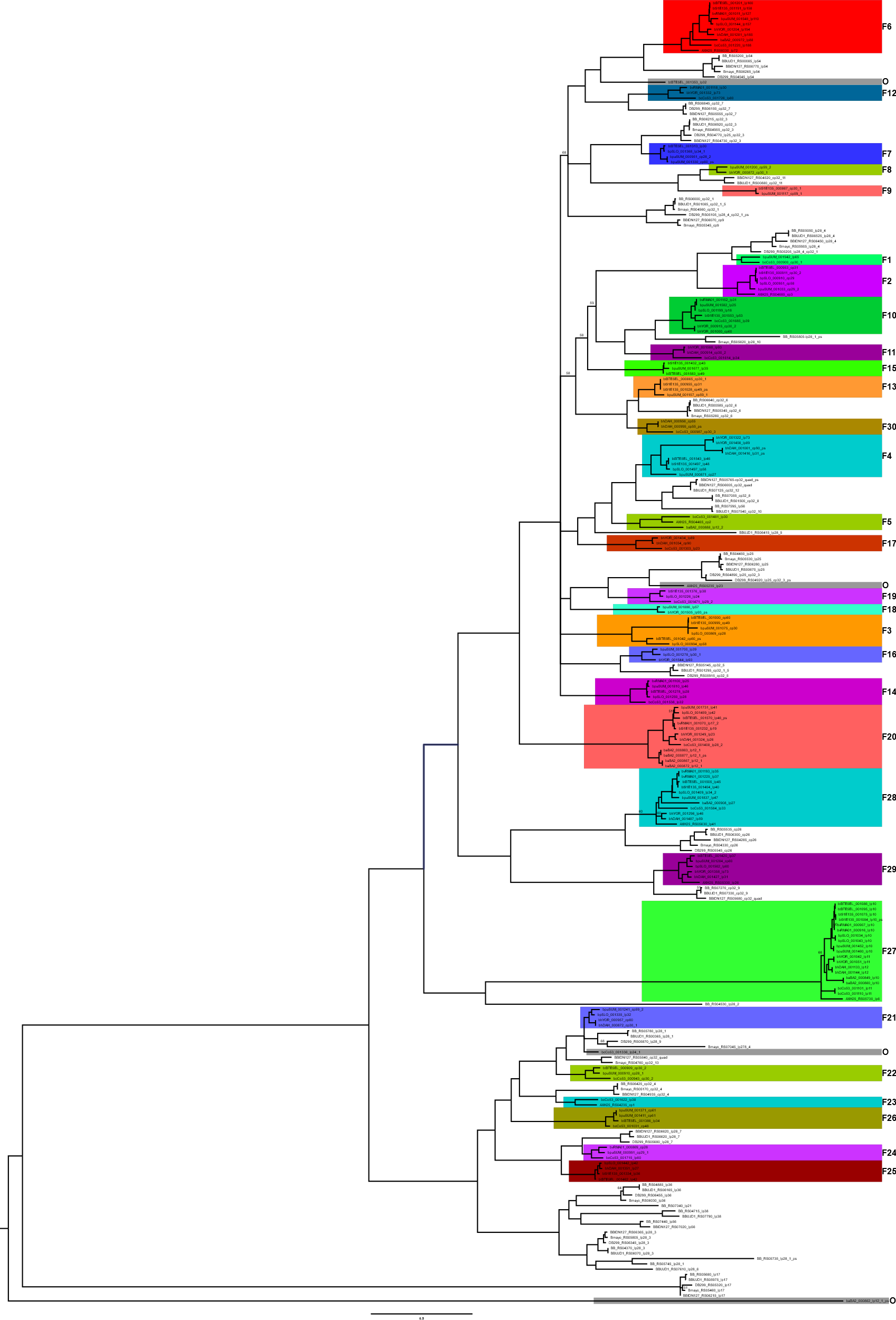


**Figure S4**

**Figure S4**. PF32 maximum-likelihood phylogenetic analysis. Maximum-likelihood tree inference was performed on the PF32 loci from the WHsTBRF spirochete, LD spirochete, and *B. miyamotoi* CT13-2396 datasets with 1,000 ultrafast bootstrap replicates. Branches with supports less than 50% were collapsed. Only the branch supports of less than 70% are shown on the tree. Plasmids that clustered were colored and given family (F) designations seen to the right of the respective cluster. Colors do not indicate similarities between families and are for ease of visualization. The locus prefix, gene locus, and plasmid are given for each PF32 gene or pseudogene (e.g. bhDAH_000872_cp30_1 indicating *B. hermsii* DAH, gene locus is 000872 found on the cp30-1 plasmid). Pseudogenes (ps) are also indicated. The scale bar indicates substitutions per site. Loci prefixes: baBA2, *B. anserina* BA2; bhDAH, *B. hermsii* DAH; bhYOR, *B. hermsii* YOR; bcCo53, *B. coriaceae* Co53; bpuSUM, *B. puertoricensis* n. sp. SUM; bpSLO, *B. parkeri* SLO; bvRMA01, *B. venezuelensis* RMA01; bt91E135, *B. turicatae* 91E135; btBTE5EL, *B. turicatae* BTE5EL; DB299, *Borreliella* (*Borrelia*) *bavarensis* Pbi; BBIDN127, *Borreliella* (*Borrelia*) *bissettii* DN12; BB, *Borreliella* (*Borrelia*) *burgdorferi* B31; BBUJD1, *Borreliella* (*Borrelia*) *burgdorferi* JD1; Bmayo, *Borreliella* (*Borrelia*) *mayonii* MN14-1420; AXH25, *Borrelia miyamotoi* CT13-2396.

**
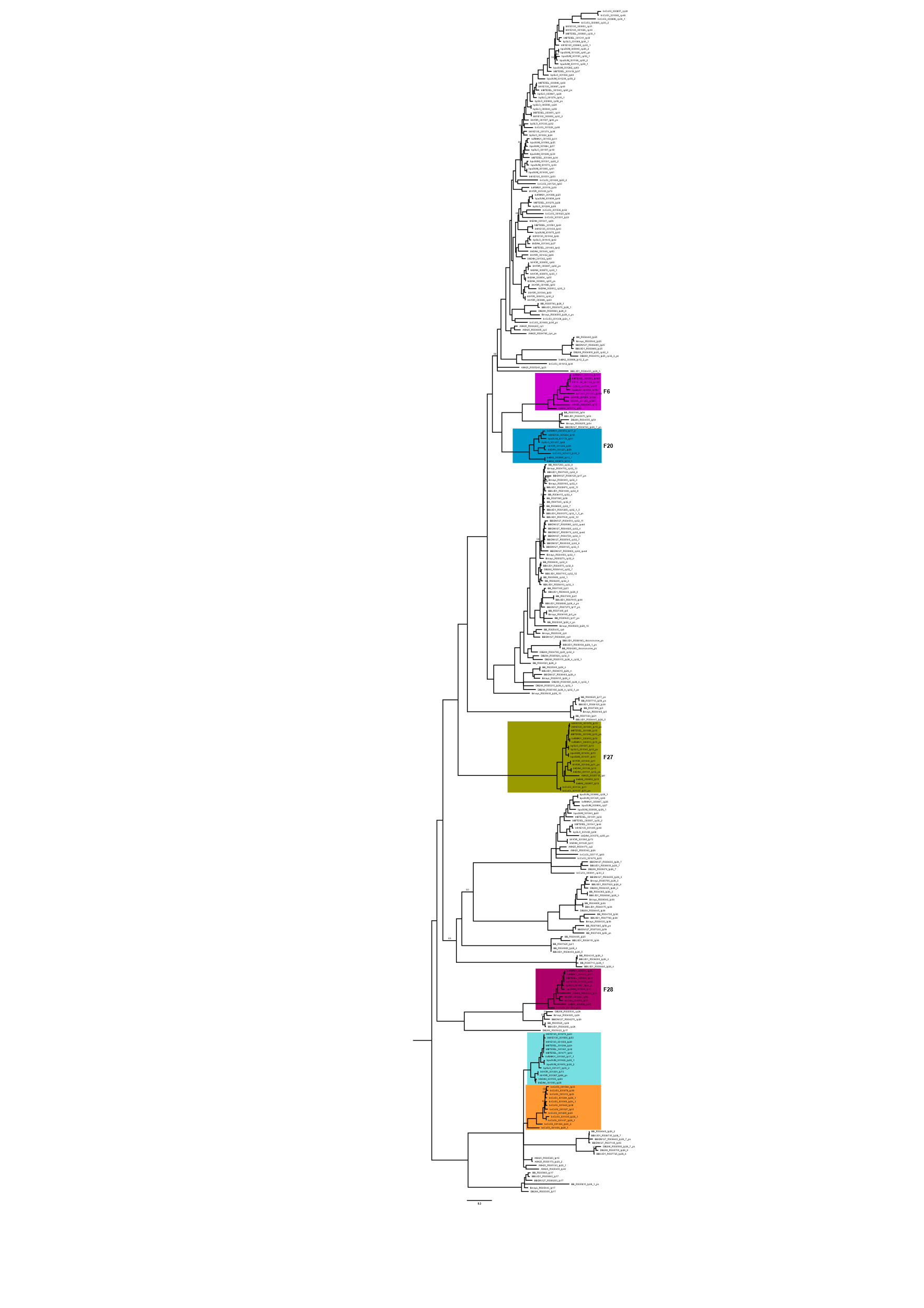
Figure S5**. PF57/62 maximum-likelihood phylogenetic analysis. A maximum-likelihood tree was inferred from the WHsTBRF spirochete, LD spirochete, and *B. miyamotoi* CT13-2396 datasets with 1,000 ultrafast bootstrap replicates. Branches with supports less than 50% were collapsed. Only the branch supports less than 70% are shown as decimals. Plasmids that clustered in agreement with the PF32 analysis were similarly colored and their PF32 family designations are indicated to the right of the respective cluster. Colors do not indicate any similarities between families/clusters and are for ease of visualization. The isolate, ORF#, plasmid is given for each PF57/62 gene/pseudogene (e.g. bhDAH_000872_cp30_1 indicating bhDAH ORF 00872 found on the cp30-1 plasmid). Pseudogenes are indicated with ‘ps’. A midpoint rooted tree was visualized in FigTree and annotated with Inkscape. The scale bar indicates substitutions per site. Loci prefixes: baBA2, *B. anserina* BA2; bhDAH, *B. hermsii* DAH; bhYOR, *B. hermsii* YOR; bcCo53, *B. coriaceae* Co53; bpuSUM, *B. puertoricensis* n. sp. SUM; bpSLO, *B. parkeri* SLO; bvRMA01, *B. venezuelensis* RMA01; bt91E135, *B. turicatae* 91E135; btBTE5EL, *B. turicatae* BTE5EL; DB299, *Borreliella* (*Borrelia*) *bavarensis* Pbi; BBIDN127, *Borreliella* (*Borrelia*) *bissettii* DN12; BB, *Borreliella* (*Borrelia*) *burgdorferi* B31; BBUJD1, *Borreliella* (*Borrelia*) *burgdorferi* JD1; Bmayo, *Borreliella* (*Borrelia*) *mayonii* MN14-1420; AXH25, *Borrelia miyamotoi* CT13-2396.

**Figure S5**


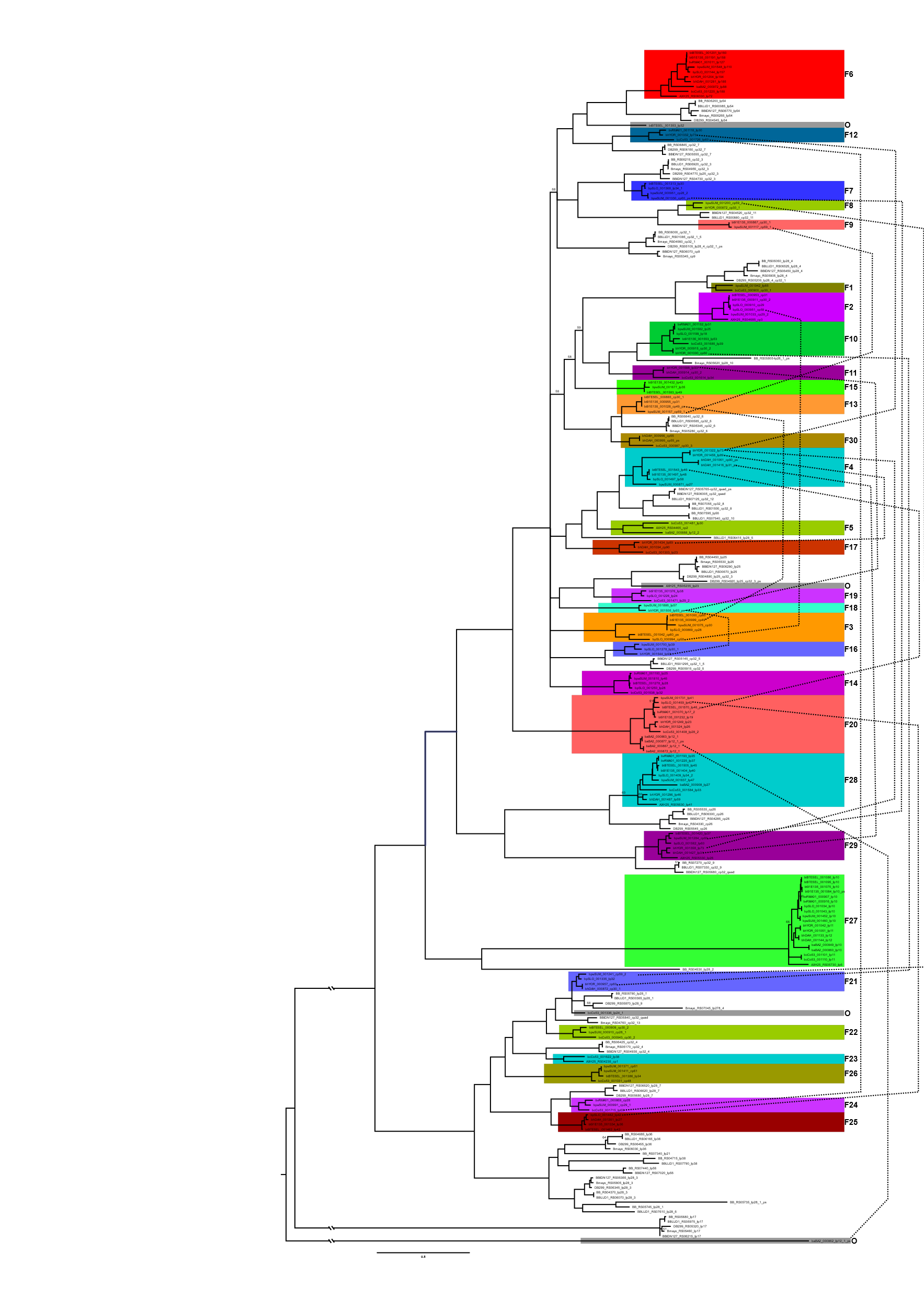


**Figure S6**

**Figure S6.** Putative plasmid fusion analysis of the PF32 phylogeny. Using the PF32 phylogeny shown in **Additional File 3: Fig S4**, the putative plasmid fusions were indicated. Dotted lines connect different WHsTBRF spirochete PF32 genes and pseudogenes found on the same plasmid of the same isolate across different plasmid clusters (e.g. bhYOR_001456_lp89 and bhYOR_001434_lp89).

**Figure S7**

**
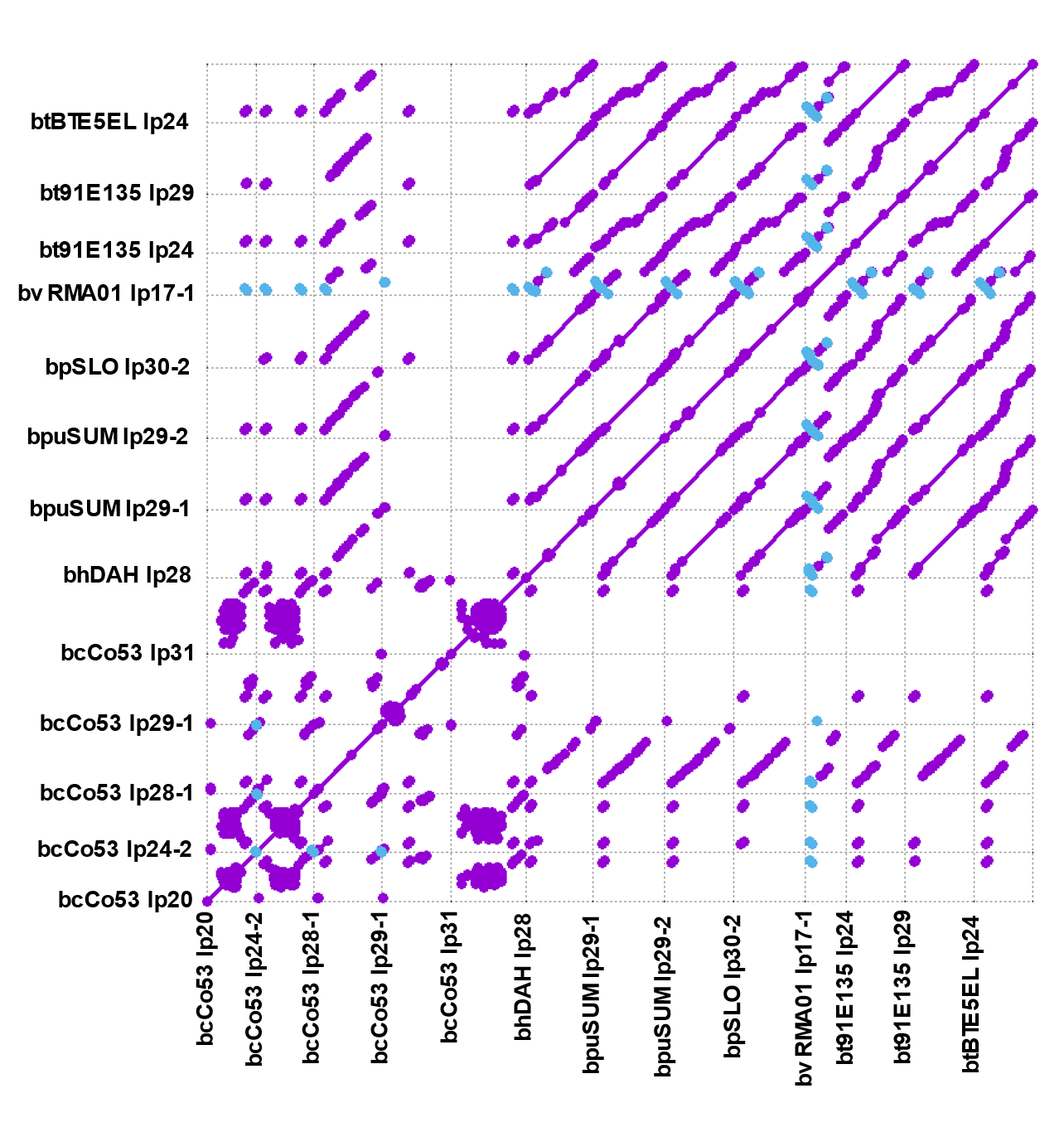
**

**Figure S7.** Dot plot analysis of WHsTBRF spirochete PF57/62-only plasmids. Dot plots were generated with Nucmer and Mummerplot using the sequences of WHsTBRF spirochete plasmids that only had the PF57/62 gene and no other plasmid partitioning gene. Sequence synteny and similarity is represented by a purple line from the bottom left corner to the top right corner of each comparison. Inversions are indicated in blue from the top left to the bottom right of each comparison.


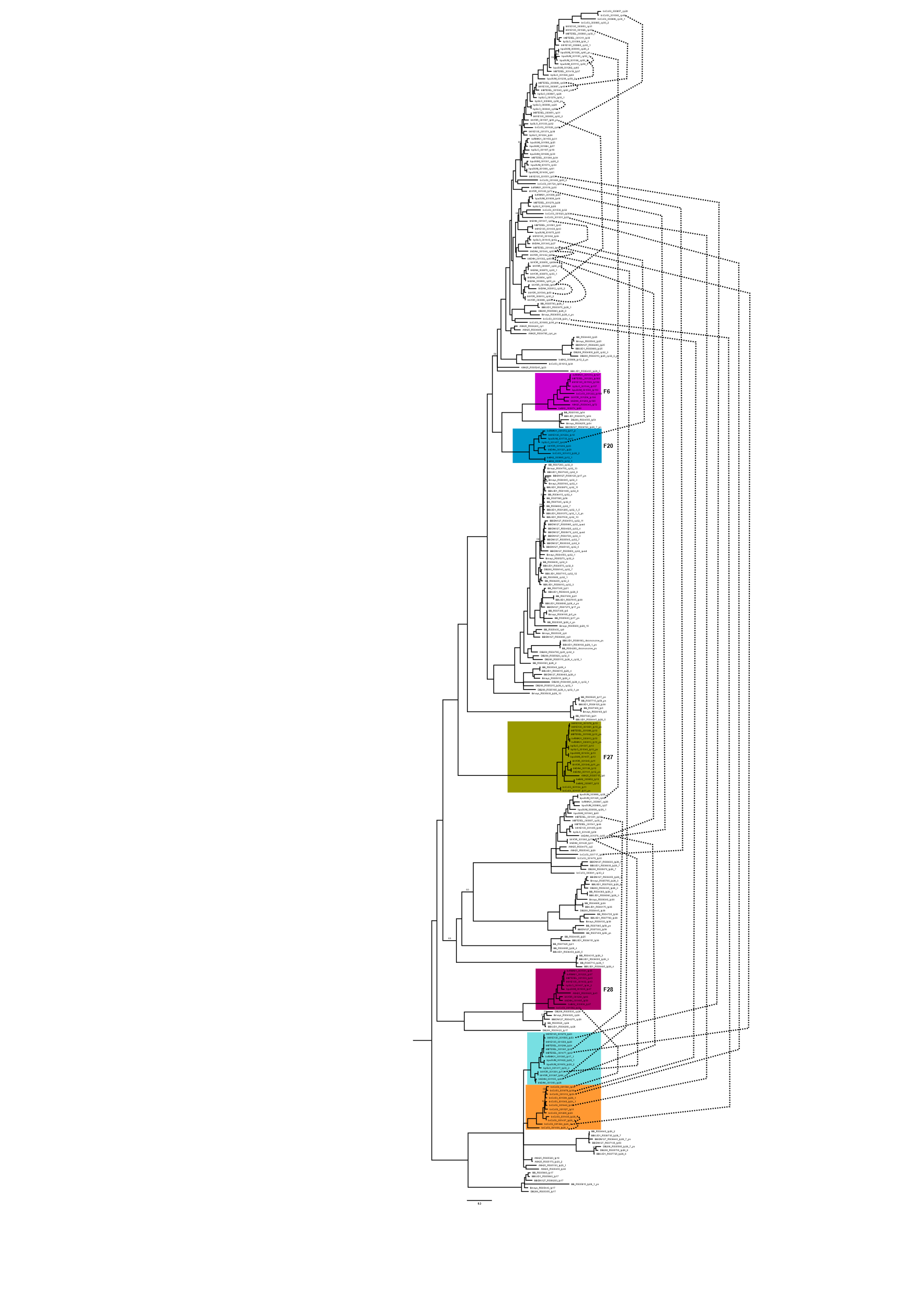


**Figure S8**

**Figure S8.** Putative plasmid fusion analysis of the PF57/62 phylogeny. Using the PF57/62 phylogeny shown in **Additional File 1:** **Fig S5**, the putative plasmid fusions were indicated. Dotted lines connect different WHsTBRF spirochete PF57/62 genes/pseudogenes found on the same plasmid across different plasmid clusters (e.g. btBTE5EL_001477_lp42 and btBTE5EL_001460_lp42).

**Figure S9**

**
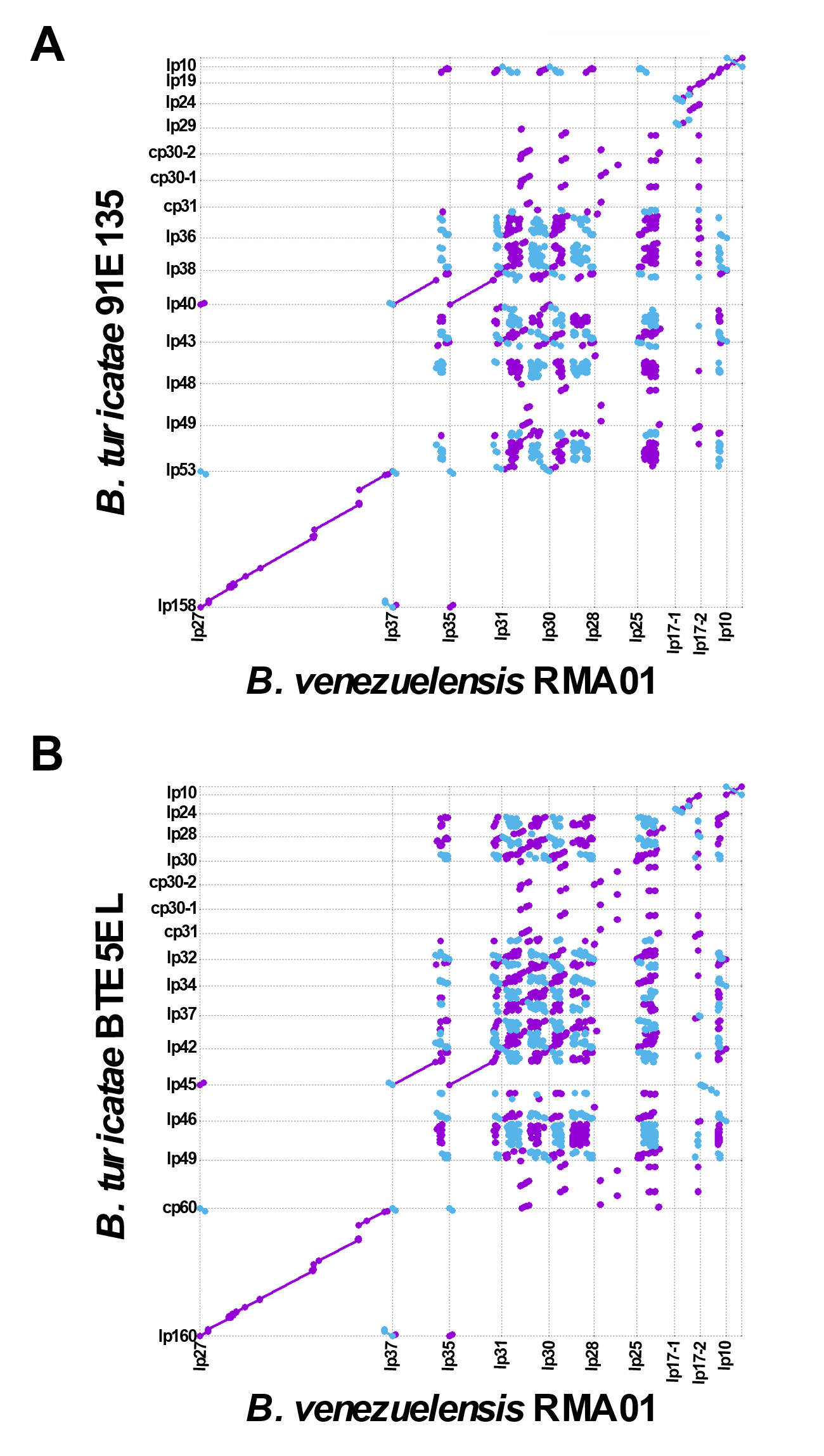
**

**Figure S9.** Dot plot comparison of plasmids of *B. venezuelensis* RMA01 and *B. turicatae* 91E135 and BTE5EL. Dot plots were generated by comparing *B. venezuelensis* RMA01 to *B. turicatae* 91E135 (**A**) and *B. turicatae* BTE5EL (**B**). Synteny is represented by a purple line from the bottom left corner to the top right corner of each comparison. Inversions are indicated in blue from the top left to the bottom right of each comparison.
